# Human iPSC-derived hepatocyte system models cholestasis with tight junction protein 2 deficiency

**DOI:** 10.1016/j.jhepr.2022.100446

**Published:** 2022-02-01

**Authors:** Chao Zheng Li, Hiromi Ogawa, Soon Seng Ng, Xindi Chen, Eriko Kishimoto, Kokoro Sakabe, Aiko Fukami, Yueh-Chiang Hu, Christopher N. Mayhew, Jennifer Hellmann, Alexander Miethke, Nahrin L. Tasnova, Samuel J.I. Blackford, Zu Ming Tang, Adam M. Syanda, Liang Ma, Fang Xiao, Melissa Sambrotta, Oliver Tavabie, Filipa Soares, Oliver Baker, Davide Danovi, Hisamitsu Hayashi, Richard J. Thompson, S. Tamir Rashid, Akihiro Asai

**Affiliations:** 1 Centre for Stem Cells and Regenerative Medicine, King’s College London, London, UK; 2 Cincinnati Children’s Hospital Medical Center, Cincinnati, OH, USA; 3 Department of Paediatrics, The University of Cincinnati, Cincinnati, OH, USA; 4 Stem Cell Hotel, King’s College London, London, UK; 5 Institute of Liver Studies King’s College London, London, United Kingdom; 6 Definigen, Cambridge, UK; 7 Genome Editing and Embryology Core Facility, King’s College London, London, UK; 8 Graduate School of Pharmaceutical Science, The University of Tokyo, Tokyo, Japan

**Keywords:** PFIC (progressive familial intrahepatic cholestasis), Cellular polarity, Bile acid transport, ALB, albumin, *ASGR2*, asialoglycoprotein receptor 2, ATP1a1, ATPases subunit alpha-1, BMP4, bone morphogenetic protein 4, BSA-FAF, bovine serum albumin fatty acid-free, BSEP, bile salt export pump, CDFDA, 5-(and-6)-carboxy-2′,7′-dichlorofluorescein, DE, definitive endoderm, DILI, drug-induced liver injury, FGF2, fibroblast growth factor 2, GCA, glycocholate, GCDCA, glycochenodeoxycholate, HCM, Hepatocyte Culture Medium, HE, hepatic endodermal, HGF, hepatocyte growth factor, HNF4a, hepatic nuclear factor 4a, iHep, iPSC-derived hepatocytes, iPSC, induced pluripotent stem cell, MDCKII, Madin–Darby canine kidney II, MRP2, multidrug resistance-associated protein 2, NTCP, Na+-TCA cotransporter, PFIC, progressive familial intrahepatic cholestasis, PI, propidium iodide, RT-qPCR, quantitative reverse transcription PCR, sgRNA, single-guide RNA, ssODN, single-stranded oligonucleotide-DNA, TCA, taurocholic acid, TCDCA, taurochenodeoxycholate, TEER, transepithelial electrical resistance, TEM, transmission electron microscopy, TJP1, tight junction protein 1, TJP2, tight junction protein 2

## Abstract

**Background & Aims:**

The truncating mutations in tight junction protein 2 (TJP2) cause progressive cholestasis, liver failure, and hepatocyte carcinogenesis. Due to the lack of effective model systems, there are no targeted medications for the liver pathology with TJP2 deficiency. We leveraged the technologies of patient-specific induced pluripotent stem cells (iPSC) and CRISPR genome-editing, and we aim to establish a disease model which recapitulates phenotypes of patients with TJP2 deficiency.

**Methods:**

We differentiated iPSC to hepatocyte-like cells (iHep) on the Transwell membrane in a polarized monolayer. Immunofluorescent staining of polarity markers was detected by a confocal microscope. The epithelial barrier function and bile acid transport of bile canaliculi were quantified between the two chambers of Transwell. The morphology of bile canaliculi was measured in iHep cultured in the Matrigel sandwich system using a fluorescent probe and live-confocal imaging.

**Results:**

The iHep differentiated from iPSC with *TJP2* mutations exhibited intracellular inclusions of disrupted apical membrane structures, distorted canalicular networks, altered distribution of apical and basolateral markers/transporters. The directional bile acid transport of bile canaliculi was compromised in the mutant hepatocytes, resembling the disease phenotypes observed in the liver of patients.

**Conclusions:**

Our iPSC-derived in vitro hepatocyte system revealed canalicular membrane disruption in TJP2 deficient hepatocytes and demonstrated the ability to model cholestatic disease with TJP2 deficiency to serve as a platform for further pathophysiologic study and drug discovery.

**Lay summary:**

We investigated a genetic liver disease, progressive familial intrahepatic cholestasis (PFIC), which causes severe liver disease in newborns and infants due to a lack of gene called TJP2. By using cutting-edge stem cell technology and genome editing methods, we established a novel disease modeling system in cell culture experiments. Our experiments demonstrated that the lack of TJP2 induced abnormal cell polarity and disrupted bile acid transport. These findings will lead to the subsequent investigation to further understand disease mechanisms and develop an effective treatment.

## Highlights

• Deficiency of tight junction protein 2 (TJP2) causes progressive cholestasis in infants, and the pathophysiology remains unclear.
• Induced pluripotent stem cells (iPSCs) from patients with TJP2 mutations were generated.
• Isogenic pairs of human iPSCs with and without TJP2 deficiency were generated by CRISPR genome editing.
• Hepatocytes derived from iPSCs with TJP2 deficiency showed impaired bile acid transport, recapitulating phenotypes of patients.
• TJP2-deficient hepatocytes exhibit canalicular membrane disruption that resulted in derangement of cellular polarity.

## Graphical abstract

**Figure:**
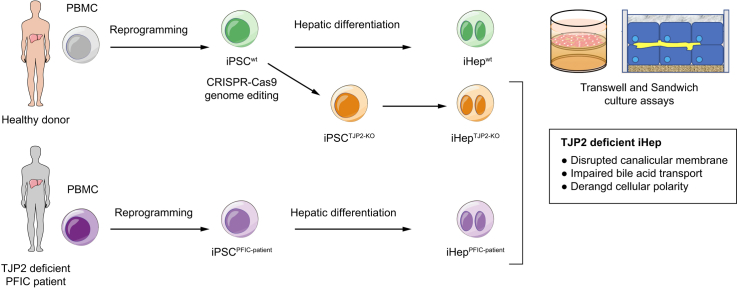

## Introduction

Recently, we and others have identified the protein-truncating mutations in the tight junction protein 2 gene (*TJP2*) to cause progressive familial intrahepatic cholestasis (PFIC).^1^^,^^2^ Newborns with TJP2 deficiency present with jaundice, pruritus, and progressive intrahepatic cholestasis and eventual liver failure and juvenile hepatocellular carcinoma.^3^ The only curative approach for PFIC with TJP2 deficiency is orthotopic liver transplantation.^1^ Because of the scarcity of organ donors, alternative therapies for patients with TJP2 deficiency are urgently needed. A clinically relevant disease model that recapitulates the pathophysiology of PFIC with TJP2 deficiency is crucial for the development of effective therapies.

The *TJP2* gene encodes TJP2 protein, also known as ZO-2. TJP2 is a cytoplasmic component of cell–cell junctional complex expressed in most epithelial cells, including hepatocytes.[4], [5], [6] In the liver, TJP2 functions as a scaffold tethering the transmembrane tight junction proteins and actin cytoskeletons to maintain the structure of bile canaliculi; thus, the tight junction separates bile from the blood.^7^ In our previous study of patients’ specimens, we found that disruption of tight junction plaques in the hepatocytes was likely caused by the lack of TJP2.^8^ Beyond the morphological observation, pathological mechanisms in the TJP2-deficient hepatocytes remain elusive.

Mouse models to study TJP2 deficiency were pursued previously.^9^ However, mice lacking TJP2 were embryonic lethal at the early developmental stage and were not feasible for mechanistic investigation. Two recent studies investigated conditional knockout mouse models by using the ALB-Cre TJP2-flox system.^10^^,^^11^ Itoh *et al.*^10^ described no significant morphological and functional changes in the Tjp2-deficient liver. Xu *et al.*^11^ showed that the Tjp2-deficient liver has minor morphological changes in the tight junction with normal permeability. They showed that the Tjp2-deficient liver has mild progressive cholestasis with lower gene expression levels of bile salt export pump (Bsep) and detoxification enzyme, Cyp2b10, which is aggravated by a cholic acid diet. In both models, mouse liver with the liver-specific knockout of Tjp2 did not recapitulate the severe neonatal cholestasis of human TJP2 deficiency. *In vitro* models using the knockdown technique on cancer cell lines were intensively used to study TJP2 deficiency in the intestinal and kidney epithelium.^12^ However, TJP2 deficiency in the hepatic epithelium was not investigated thoroughly because of the lack of sufficient models. Therefore, we sought to apply novel technologies of pluripotent stem cells, based on the recent success in using human induced pluripotent stem cells (iPSCs) to build a hepatic epithelial culture system[13], [14], [15] and using iPSCs from patients with similar genetic cholestatic disorders to study the impact of *ABCB11* and alpha-1-antitrypsin (*SERPINA1*) mutations on hepatocytes.^16^^,^^17^ Additionally, we also sought to apply the CRISPR/Cas9 genome-editing technology to elucidate the monogenic effect of TJP2 deficiency in this study.

We hypothesised that iPSC-derived hepatocytes (iHep) serve as a platform to interrogate the disease mechanisms in hepatocytes and investigate the genotype–phenotype association and aimed to demonstrate that the *TJP2* mutations cause cholestatic liver disease by impairing the ability of hepatocytes to generate functional bile canaliculi.

## Materials and methods

### Human iPSC reprogramming and culture

Human specimens were obtained from patients according to ethically approved protocols (08/H0311/201 and 08/H0712/34+5-IRAS project ID: 134561 for King’s College Hospital and Study ID: 2014-6705 the Institutional Review Board of Cincinnati Children’s Hospital Medical Center). About 8-mm skin punch biopsies or peripheral blood cells were collected from volunteer patients attending King’s College Hospital or Cincinnati Children’s Medical Center with informed consent. Fibroblasts were derived from the donor tissue in GMP conditions using standardised protocols. Nonintegrating OriP/EBNA1 vectors containing 5 reprogramming factors (Oct4, Sox2, Lin28, Klf4, and L-Myc) were transfected into the patient fibroblasts as previously described.^18^ The entire reprogramming process was feeder cell-free. Successful human iPSC clone was cultured in Essential 8™ Medium or mTeSR-1 specially formulated for the growth and expansion of human pluripotent stem cells.

### Pairs of isogenic iPSC generation with CRISPR-CAS9 genomic editing

The control iPSCs (clone code: 1383D6) were derived from a healthy donor and provided by Kyoto University with a thorough characterisation of pluripotency and karyotype.^19^ iPSC lines were engineered with CRISPR-CAS9 technology by introducing mutations of TJP2 into parental iPSCs obtained from healthy patients. Genome editing to obtain homozygous c.1099C>T, p.367X mutation in *TJP2* was achieved by selecting candidate single-guide RNA (sgRNA) from CRISPOR (http://crispor.org/) and cloning into the pX458M-HF vector that was modified from the pX458 vector (addgene #48138) and a high-fidelity Cas9 (eSpCas9 1.1)-2A-GFP expression cassette. The editing activity of the plasmid was validated in 293T cells by T7E1 assay.[20], [21], [22] A phosphorothioated single-stranded oligonucleotide-DNA (ssODN) was designed to include the intended mutations and silent mutations (to block sgRNA retargeting and to create a new restriction enzyme site for genotyping). A single-cell suspension of iPSCs was prepared using Accutase, and 1×10^6^ cells were nucleofected with 2.5 μg of the plasmid and 2.5 μg of ssODN using program CA137 (Lonza). Then, 48 h later, transfected cells were sorted 1 cell per well into 96-well plates based on the GFP expression. The correctly edited clones were selected based on the loss of the restriction enzyme sites on both alleles and further confirmed by Sanger sequencing for the identification of bi-allelic single nucleotide deletion. Cell clones that went through the same targeting process but remained unedited were expanded and used as isogenic parental controls.

### iPSC to iHep differentiation

A Transwell-based hepatocyte differentiation protocol was used in this study, which was modified from previously described protocols.^13^ Briefly, for definitive endodermal (DE) differentiation, iPSCs were dissociated with Accutase and plated onto a Laminin 511 (Matrixsome)-coated cell culture dish. The medium was replaced with RPMI 1640 (ThermoFisher) containing 2% B27 (ThermoFisher), 0.5 mM sodium butyrate, Wnt3a 50 ng/ml (R&D Systems, Minneapolis, MN, USA) and Activin 100 ng/ml (R&D Systems) for 6 days. For hepatic specification, cells were further treated with fibroblast growth factor 2 (FGF2) 10 ng/ml (R&D Systems) and bone morphogenetic protein 4 (BMP4) 20 ng/ml (R&D Systems) for 3 days. Cells were dissociated with TrypLE (ThermoFisher) and were plated on the membrane of the Transwell insert (Corning). Then, cells were cultured in Hepatocyte Culture Medium (HCM; Lonza) for 12 days. HCM was supplemented with HCM BulletKit (Lonza): transferrin, hydrocortisone, bovine serum albumin fatty acid-free (BSA-FAF), ascorbic acid, insulin, GA-1000, and omitting human epidermal growth factor. In addition, 10 ng/ml recombinant hepatocyte growth factor (HGF), 100 nM dexamethasone, and 5% of FBS (ThermoFisher) were added to supplement HCM. HGF was removed from the medium 3 days before the experiments when indicated. We also used a Matrigel (Corning) sandwich-based hepatocyte differentiation protocol for the quantification of the bile canalicular network, modified from the previous publication.^23^ Human iPSCs were passaged using gentle cell dissociation reagents (STEMCELL Technologies) and then transferred onto plates precoated with gelatine. Cells were then grown in 3 different culture media, Essential 6, RPMI, and Hepatozyme supplemented with 7 different differentiation factors, namely, CHIR99021 (3 μM; Stemgent), Ly294002 (10 μM; Calbiochem), Activin (100 ng/ml; R&D Systems), FGF2 (40 ng/ml; R&D Systems), BMP4 (10 ng/ml; R&D Systems), HGF (20 μg/ml; PeproTech), and Oncostatin-M (10 μg/ml; R&D Systems). For cells to establish polarity in the Matrigel sandwich, on Day 21 of hepatic differentiation, 6% Matrigel (Corning) was overlaid onto iHep cultured in a 96-well plate for 1 week.

### CDFDA functional assay in Matrigel sandwich

To evaluate the bile acid transport capacity and morphology of bile canaliculi in iHep, multidrug resistance-associated protein 2 (MRP2)-specific substrate 5-(and-6)-carboxy-2′,7′-dichlorofluorescein (CDFDA) was supplemented into cells for 20 min. Accumulation of fluorescent tracer (a metabolite of CDFDA) in bile canaliculi was then captured by CLS high content confocal imaging (Operetta CLS) and analysed using ImageJ software. The images were first processed using global thresholding to filter out the noise and saturated signals. Next, size exclusion filtering was applied to remove imaging artefacts and to select biological relevant signals to determine the area, circularity, Feret’s diameter, aspect ratio, roundness, and solidarity of the accumulated fluorescent tracer in bile canaliculi.

## Results

### Generation of patient-specific iPSCs and CRISPR-edited iPSCs

**Fig. 1:**
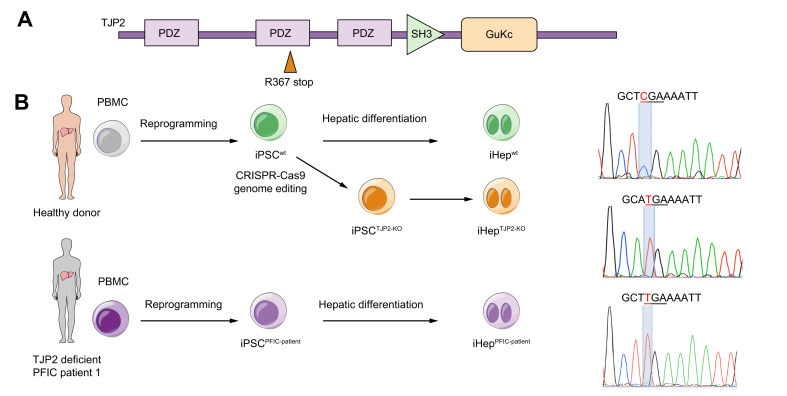
Generation of iPSC lines with *TJP2* truncating mutations from patient’s cells and CRISPR-CAS9 genome-editing technology. (A) Diagrams illustrating the location of *TJP2* mutation (c.1099C>T) and protein truncation of TJP2 (p.R367X). PDZ, SH3, and GuKc are functional domains of TJP2. (B) Schematic illustration of the roadmap of human iPSC-derived hepatocytes modelling PFIC with TJP2 deficiency. An iPCS^TJP2-KO^ line was generated using the CRISPR-CAS9 genome-editing technology to introduce truncating mutations into the *TJP2* gene in the iPSC^wt^, which was originally obtained from a healthy donor. An iPSC^PFIC-patient^ line was generated from a patient with PFIC with TJP2 deficiency. Morphological and functional assays were performed after inducing hepatic differentiation into each iPSC (iHep). The genotypes were confirmed by Sanger sequencing of the c.1099 locus. GuKc, guanylate kinase homologous; iHep, iPSC-derived hepatocytes; iPSC, induced pluripotent stem cell; PBMC, peripheral blood mononuclear cells; PDZ, Psd-95 (post synaptic density protein), DlgA (drosophila disc large tumor suppressor) and ZO1 *(zonula occludens-1 protein)*; PFIC, progressive familial intrahepatic cholestasis; SH3, Src homology 3; TJP2, tight junction protein 2.

As a prototype of this study, we focused on 1 patient who developed PFIC from homozygotic truncating mutations of TJP2 (p.R367X) (Fig. 1A). We first generated an iPSC line from this patient by reprogramming her peripheral blood cells (iPSC^PFIC-patient^) and iPSC lines from healthy donors to serve as control lines (iPSC^wt^). Then, to enhance the reproducibility of our study by comparing an isogenic pair, we generated a daughter iPSC line from iPSC^wt^ using CRISPR-Cas9 technology to introduce the same homozygotic p.R367X truncation (iPSC^TJP2-KO^). This set of 3 iPSC lines were then differentiated into hepatocytes (iHep) to test whether they model the phenotype of TJP2 deficiency (Fig. 1B). We confirmed the genotypes of generated iPSCs by Sanger sequencing. Each iPSC line in the set of 3 iPSCs (iPSC^wt^, iPSC^TJP2-KO^, and iPSC^PFIC-patient^) exhibited c.1009C (reference sequence), c.1009T (p.R367X), and c.1009T (p.R367X), respectively. Then, we generated another set of iPSC lines to study another genotype, homozygous truncating mutations in *TJP2* (Y261Sfs∗50: truncating at 310), found in a patient with PFIC (Fig. S1A). The Sanger sequencing of the second set of iPSCs also exhibited truncating mutations equivalent to the mutation found in the patient (Fig. S1B). Collectively, we have successfully generated patient-derived and CRISPR-edited iPSC lines with *TJP2* mutations reproducing the patients’ genotypes. We listed iPSC lines used in the study in the Supplementary information tables.

### *TJP2* mutations induced TJP2 deficiency in hepatocyte-like cells while maintaining the comparable ability of hepatic differentiation

**Fig. 2:**
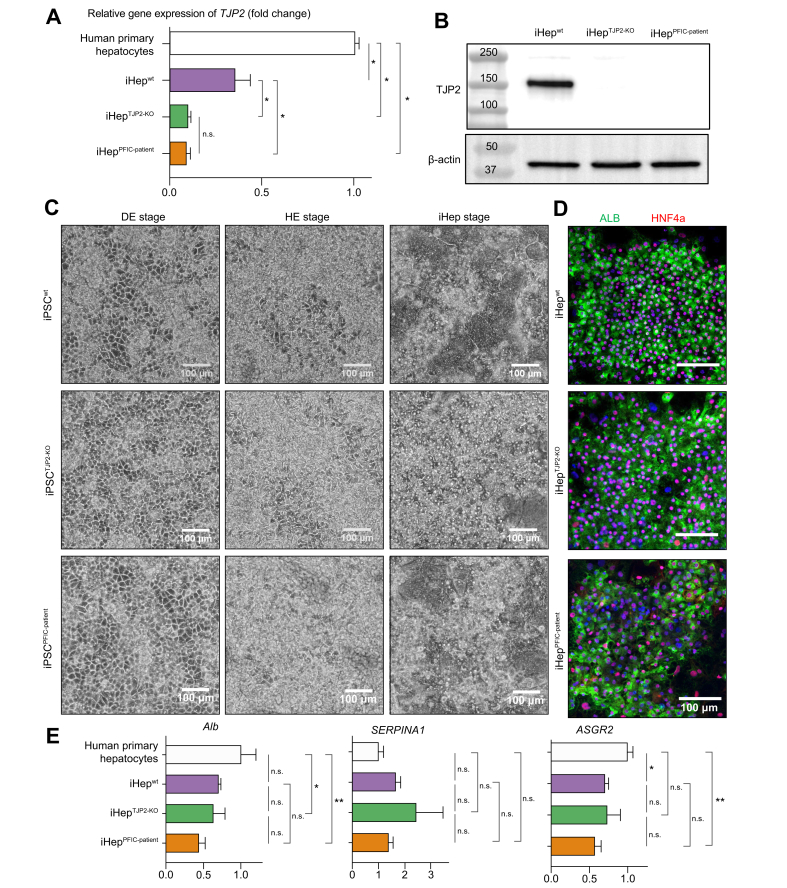
Characterisation of the human hepatocyte-like cells differentiated from iPSCs. (A) Quantitative gene expression analysis by RT-qPCR reveals that the *TJP2* is significantly downregulated in hepatocyte-like cells differentiated from iPSC^TJP2-KO^ and iPSC^PFIC-patient^ (iHep^TJP2-KO^ and iHep^PFIC-patient^), compared with iHep^wt^. Mean ± SD, n = 3, where n corresponds to independent experiments. One-way ANOVA with *post hoc* analysis. ∗*p* <0.01, n.s. (B) Western blotting reveals that TJP2 protein is absent in the iHep with *TJP2* mutation (iHep^TJP2-KO^ and iHep^PFIC-patient^). Beta-actin serves as a loading control. (C) Brightfield microscopy images reveal the morphological changes of each iPSC line at each stage of differentiation. Scale bar: 100 μm. (D) Immunofluorescence images detect the signature markers of hepatocytes (HNF4a: red, ALB: green, and Nuc: blue), revealing a similar pattern of hepatic maturation in iHep^wt^, iHep^TJP2-KO^, and iHep^PFIC-patient^. (E) RT-qPCR gene expressions of key hepatic genes (*ALB, SERPINA1,* and *ASGR2*) are comparable among iHep^wt^, iHep^TJP2-KO^, and iHep^PFIC-patient^. Relative gene expression was displayed as fold changes by −ΔΔCt method using 18S as a housekeeping gene and the expression level of human primary hepatocytes as a relative baseline. One-way ANOVA with *post hoc* analysis, ∗*p* <0.05, ^†^*p* <0.01, ns. Mean ± SD, n = 3, n corresponds to independent experiments. ALB, albumin; *ASGR2*, asialoglycoprotein receptor 2; DE, definitive endoderm; HE, hepatic specification; HNF4a, hepatic nuclear factor 4a; iHep, iPSC-derived hepatocytes; iPSC, induced pluripotent stem cell; ns, nonsignificant; PFIC, progressive familial intrahepatic cholestasis; RT-qPCR, quantitative reverse transcription PCR; TJP2, tight junction protein 2.

To study the impact of *TJP2* mutations on hepatocytes, we induced hepatic differentiation into all iPSC lines, using our previously developed protocols that mimic the stages of human liver development.^13^^,^^23^ The set of iPSC lines (iPSC^wt^, iPSC^TJP2-KO^, and iPSC^PFIC-patient^) were used to optimise the hepatocyte differentiation protocols to generate hepatocyte-like cells (iHep^wt^, iHep^TJP2-KO^, and iHep^PFIC-patient^, respectively). The iPSCs were incubated with culture medium with defined growth factors and formed a confluent monolayer by DE stage and hepatic endodermal (HE) stage. Further maturation to hepatocyte-like cells (iHep) was induced with Oncostatin-M and HGF in the media. First, *TJP2* gene expression of iHep was compared by quantitative reverse transcription PCR (RT-qPCR). iHep^PFIC-patient^ and iHep^TJP2-KO^ showed significantly lower *TJP2* expression compared with iHep^wt^ (Fig. 2A and Fig. S2A). Western blot further demonstrated that TJP2 protein is deficient in iHep^TJP2-KO^ and iHep^PFIC-patient^ (Fig. 2B). We monitored the morphological transformation of cells throughout the differentiation stages (Fig. 2C and Fig. S2B). Immunofluorescence staining revealed that iHep expresses hepatocyte-specific markers such as hepatic nuclear factor 4a (HNF4A) and albumin (ALB), further suggesting that all iPSC lines were capable of differentiating into hepatocyte-like cells (Fig. 2D and Fig. S2C). The gene expression of the hepatic-specific genes, *ALB*, asialoglycoprotein receptor 2 (*ASGR2*), and *SERPINA1*, in iHep were comparable among lines (Fig. 2E and Fig. S2D). Collectively, our data demonstrated that premature stop codons in the sequence of *TJP2* gene caused protein deficiency of TJP2 in induced hepatocytes. We also showed that all iPSC lines after genome editing were capable of differentiating into iHep with comparable expression levels of hepatic differentiation markers.

### TJP2 deficiency induces disruption of the apical canalicular membrane and inclusion bodies

**Fig. 3:**
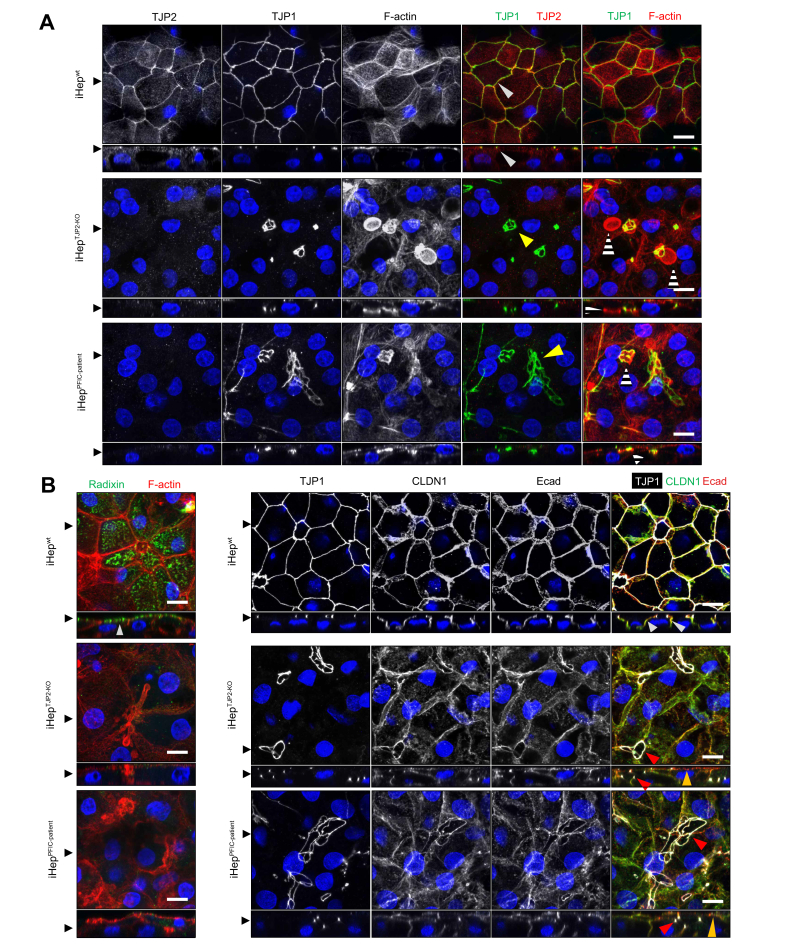
TJP2 deficiency in iHep alters distribution of apical and basolateral markers. (A) Orthogonal view from different planes (*x*/*y* and *x*/*z*) of the confocal micrographs shows the expression of tight junction proteins detected in iHep^wt^, iHep^TJP2-KO^, and iHep^PFIC-patient^ cultured in the Transwell system. In the left 3 columns, DAPI (blue) was overlayed on the grayscale images of TJP1, TJP2, and F-actin. In the right 2 columns, TJP1 (green) and TJP2 (red) or TJP1 (green) and F-actin (red) were overlayed. In iHep^wt^, TJP1 and TJP2 colocalise at the tight junction of adjacent cells (white arrowheads). In iHep^TJP2-KO^ and iHep^PFIC-patient^, TJP1 is detected in circular forms with random patterns (yellow arrowheads), and TJP2 is not detected. F-actin positive disc-like structures (white/black stripes) were found in iHep^TJP2-KO^ and iHep^PFIC-patient^. The disc-like structures are located at lower levels than the apical membrane. The black arrowheads on the side of images indicate the position of Z-stacking and *x*/*y* sections. Scale bar: 10 μm. (B) Orthogonal views from different planes (*x*/*y* and *x*/*z*) of the confocal micrographs of iHep^wt^, iHep^TJP2-KO^, and iHep^PFIC-patient^ cultured in the Transwell culture system display immunofluorescent staining of cell polarity markers. Radixin (green) localised on the apical membrane in iHep^wt^ (white arrowhead) but not detected in iHep^TJP2-KO^ and iHep^PFIC-patient^. TJP1 was used as a landmark of tight junctions. Z-stack analysis of iHep^wt^ showed that CLDN1 (a tight junction and basolateral membrane marker; grayscale and green) and Ecad (a basolateral membrane marker; grayscale and red) are localised on the basolateral membrane (white arrowheads). In iHep^TJP2-KO^ and iHep^PFIC-patient^, Ecad is localised on the basolateral membranes and apical membranes (orange arrowheads). The circular pattern of TJP1 expression (red arrowheads) is overlapped with CLDN1 and Ecad expression, but Radixin was absent. Scale bar: 10μm. CLDN1, Claudin1; Ecad, Ecadherin; iHep, iPSC-derived hepatocytes; iPSC, induced pluripotent stem cell; PFIC, progressive familial intrahepatic cholestasis; TJP1, tight junction protein 1; TJP2, tight junction protein 2.

**Fig. 4:**
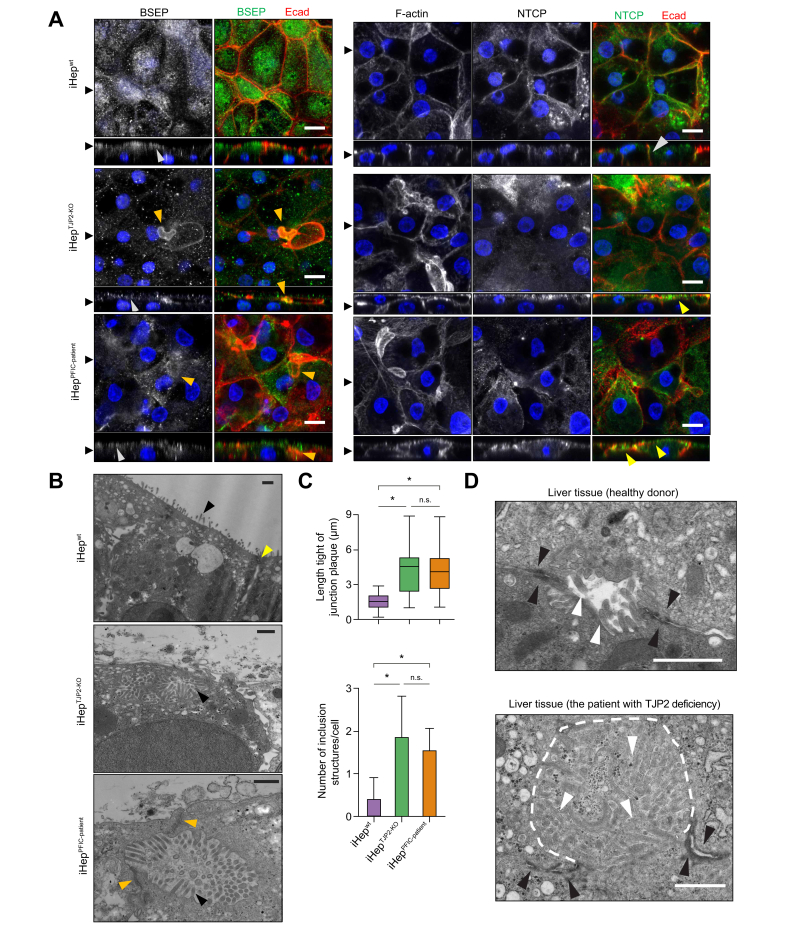
TJP2 deficient iHep exhibit apical membrane disruption. (A) Reconstructed cross-section (*x*/*z* plane) of the confocal micrographs show the BSEP (an apical transporter; left panels in grayscale and green) and NTCP (a basolateral transporter; right panels in grayscale and green) of iHep^wt^, iHep^TJP2-KO^, and iHep^PFIC-patient^ cultured in the Transwell culture system. BSEP is localised on the apical membrane (white arrowheads) in all iHep and F-actin positive spherical/disc-like structures found in iHep^TJP2-KO^ and iHep^PFIC-patient^ (orange arrowheads). NTCP is localised on the lateral membrane in iHep^wt^ (white arrowheads), whereas it is localised on the apical membrane and cytoplasm in iHep^TJP2-KO^ and iHep^PFIC-patient^ (yellow arrowheads). Of note, the basal membrane is not visualised because of the optical interference of the plastic membrane of the Transwell. Scale bar: 10 μm. (B) Ultrastructural images of transmission electron micrographs (TEM) of iHep^wt^ show the apical membrane covered with microvilli (black arrowheads) and tight junction plaques were found between cells (yellow arrowheads). In iHep^TJP2-KO^ and iHep^PFIC-patient^, inclusion structures containing the apical microvilli (black arrowheads) were found. (C) Morphometric analysis revealed elongated tight junction plaques and an increased number of inclusion structures in iHep^TJP2-KO^ and iHep^PFIC-patient^. One-way ANOVA with *post hoc* analysis, ∗*p* <0.05, ns. Mean ± SD, 10 cells in each genotype per experiment and n = 3, where n corresponds to independent experiments. (D) The liver specimen from a patient without liver disease show intact bile canaliculus that is found between hepatocytes, sealed by tight junction plaques (black arrowheads). The canalicular lumen is rich in membranous protrusions of microvilli (white arrowheads). In the TEM image from the patient with TJP2 mutations (R367X), a disrupted apical membrane with microvilli (white arrowheads) and an inclusion structure (white dashed lines) are found. Disrupted tight junction plaques (black arrowheads) are shown. Scale bars: 2 μm. BSEP, bile salt export pump; Ecad, Ecadherin; iHep, iPSC-derived hepatocytes; iPSC, induced pluripotent stem cell; ns, nonsignificant; NTCP, Na+-taurocholic acid cotransporter; PFIC, progressive familial intrahepatic cholestasis; TEM, transmission electron microscopy; TJP2, tight junction protein 2.

Bile canalicular formation is a hallmark of the maturation of hepatocytes in the liver. The apical membranes of bile canaliculi are composed of microvilli and perform transmembrane transport of bile acid from the intracellular domain of hepatocytes into the extracellular bile canalicular domain. We hypothesise that TJP2 deficiency causes cholestasis by disrupting the formation of functional bile canaliculi. To test this hypothesis, we induced hepatic differentiation into the iPSC in the Transwell culture system to facilitate hepatocytic polarity *in vitro*. We assessed the localisation of tight junction and apical or basolateral marker proteins that signify the bile canalicular formation. For the healthy control (iHep^wt^), immunofluorescent staining revealed that tight junction protein 1 (TJP1), the main protein that is necessary for tight junctions, was localised at the cell–cell junction, forming a fishnet-like pattern, which is consistent with our previous studies (Fig. 3A and Fig. S3A).^13^^,^^23^ TJP1 localisation is limited to the corner of cells where the apical and lateral membranes separate. In addition to colocalisation with TJP1, TJP2 is also detected at the cytoplasm and the apical membrane. This pattern of TJP1 and TJP2 localisation was abrogated in the TJP2-deficient lines (iHep^TJP2-KO^ and iHep^PFIC-patient^). Although the TJP1 expression was still captured in the staining, albeit the disorganised structure, the TJP2 expression was absent, recapitulating histochemical staining on the liver of patients with PFIC with *TJP2* mutation.^1^ To further depict cellular polarity affected by TJP2 deficiency, we detected the cellular location of Ecadherin (a basolateral marker), Radixin (an apical marker), Claudin1 (a tight junction marker), and F-actin (a relative apical marker of the polarised hepatocytes; Fig. 3B). In iHep^wt^, Ecadherin, Radixin, TJP1, Claudin1, and F-actin exhibited distinctive apical–basolateral polarised distribution. However, this apical–basal pattern of Ecadherin and Radixin was lost in iHep^TJP2-KO^ and iHep^PFIC-patient^. F-actin was clustered into small spherical structures, which contain linear circles positive for TJP1, Claudin1, and Ecadherin. A basolateral marker, beta-catenin, was deranged (Fig. S3B), and a basolateral transporter, Na+/K+-transporting ATPases subunit alpha-1 (ATP1a1), was reduced and deranged (Fig. S3C). The distortion in structural polarity observed in the TJP2-deficient cells (iHep^TJP2-KO^ and iHep^PFIC-patient^) coincides with disrupted localisation of functional hepatic transporters, as demonstrated by the staining pattern of bile salt export pump (BSEP) and Na+-taurocholic acid (TCA) cotransporter (NTCP) (Fig. 4A). The reconstructed orthogonal Z-stack images revealed that iHep^wt^ display the apical membrane localisation of BSEP and lateral localisation of NTCP (basal staining was not detectable because of the optical interference of the confocal microscope), whereas iHep^TJP2-KO^ and iHep^PFIC-patient^ exhibited localisation of BSEP in the spherical structures and the apical localisation of NTCP, suggesting the bile canalicular transporting function is impaired in TJP2-deficient hepatocytes.

The ultrastructural analyses by transmission electron microscopy (TEM) further revealed the structural complexity of the bile canalicular structure in iHep (Fig. 4B). The overall architecture of the apical membrane with microvilli was established in iHep^wt^. In iHep^TJP2-KO^ and iHep^PFIC-Patient^, intracellular inclusion structures containing the microvilli of the apical membrane were found. These inclusions with the apical membrane might be the part of spherical structures found in immunofluorescent staining of iHep^TJP2-KO^ and iHep^PFIC-Patient^ because F-actin marks microvilli. Morphometric analysis of TEM images among iHep^wt^, iHep^TJP2-KO^, and iHep^PFIC-Patient^ revealed that the number of microvilli containing inclusion bodies and the length of tight junction plaques between cells were increased in iHep^TJP2-KO^ and iHep^PFIC-Patient^ (Fig. 4C). To determine whether the apical membrane disruption occurs in the human liver with TJP2 deficiency, we investigated liver tissues of patients using TEM (Fig. 4D). In a healthy liver, the bile canaliculi were found between hepatocytes, sealed by tight junction plaques (black arrowheads). The canalicular lumen, clamped by tight junction plaques, is rich in membranous protrusions of microvilli (marked by white arrowheads), signifying mature bile canaliculi. TEM images of the patient’s liver biopsy revealed a distorted bile canaliculus with an inclusion structure containing the apical membrane (white dotted lines) between the distorted tight junction plaques (black arrowheads), showing significant morphological similarity found in iHep^TJP2-KO^ and iHep^PFIC-Patient^. These findings suggest that TJP2 deficiency induces the apical membrane disruption and derangement of cellular polarity in hepatocytes.

**Fig. 5:**
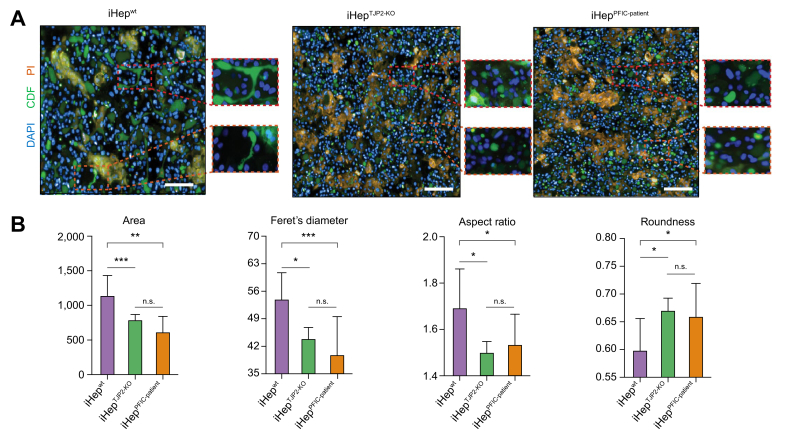
A morphometric assay on Sandwich cultured iHep demonstrates disturbed bile canalicular networks in TJP2 deficiency. (A) Representative confocal micrographs of iHep^wt^, iHep^TJP2-KO^, and iHep^PFIC-patient^ were taken by a high-throughput imaging system, Operetta CLS™, show the transport and accumulation of a fluorescent tracer (CDF: 5-(and-6)-carboxy-2’,7’-dichlorofluorescein, metabolites of CDFDA, green) representing the function and morphology of bile canaliculi of cells cultured in Matrigel sandwich system. Dead cells are stained by PI (red), and nuclei are stained by DAPI (blue) to assess the viability of cells. iHep^wt^ displayed a chicken-wire-like network of bile canaliculi, whereas iHep^TJP2-KO^ and iHep^PFIC-patient^ displayed small spherical bile canalicular structures accumulating fluorescent tracers. Scale bar, 100 μm. (B) Morphological quantification of bile canalicular network and structure observed in CDFDA assays using the ImageJ processing algorithm. The pattern of fluorescent tracers in iHep^TJP2-KO^ and iHep^PFIC-patient^ showed significantly smaller, less branching (Feret’s diameter) and more round structures compared with that in iHep^wt^. Mean ± SD, n = 9, where n corresponds to independent experiments, unpaired Student *t* test, ∗*p* <0.05, ^†^*p* <0.01, ^‡^*p* <0.001, and ns. CDFDA, 5-(and-6)-carboxy-2′,7′-dichlorofluorescein; iHep, iPSC-derived hepatocytes; iPSC, induced pluripotent stem cell; ns, nonsignificant; PFIC, progressive familial intrahepatic cholestasis; PI, propidium iodide; TJP2, tight junction protein 2.

Further, to determine whether these disrupted apical membranes compromise the bile canalicular network in TJP2-deficient hepatocytes, we used carboxyfluorescein diacetate succinimidyl ester (CDFDA), a fluorescent tracer of a hepatic apical transporter (MRP2), to visualise the extension of bile canaliculi in Matrigel-sandwich cultured iHep. In iHep^wt^, the active transport and accumulation of the fluorescent tracer were detected along the border of adjacent cells and formed elongating network of bile canaliculi (Fig. 5A). The morphology of the bile canaliculi was significantly different in TJP2-deficient iHep. The bile canalicular network that resembles a chicken wire-like structure established in the healthy (iHep^wt^) was lost in the iHep^PFIC-patient^ and iHep^TJP2-KO^. Instead, the small and isolated spherical structures were revealed, as shown in the inlets. To quantify the bile canalicular network structure by morphometric analysis, we developed an image-processing assay to evaluate the transport and accumulation of the tracer captured by a high-throughput confocal microscopy system. The morphometric analysis of bile canaliculi in TJP2-deficient iHep revealed that they are smaller (area), less branching (Feret’s diameter), less elongated (aspect ratio), and more swelled in shapes (roundness) (Fig. 5B). The viability of iHep, measured by propidium iodide (PI)-positive nuclei, was comparable among groups (Fig. S4).

Collectively, iHep^TJP2-KO^ and iHep^PFIC-patient^ show the lack of TJP2 expression and significant structural distortion on the cellular polarity and the bile canalicular network compared with the iHep^wt^. This coincides with disrupted localisation of functional transporters, suggesting that TJP2-deficient iHep recapitulate the pathology occurring in the liver of patients with PFIC with *TJP2* mutation.

### *TJP2* mutations in iHep induce impaired bile canalicular function

**Fig. 6:**
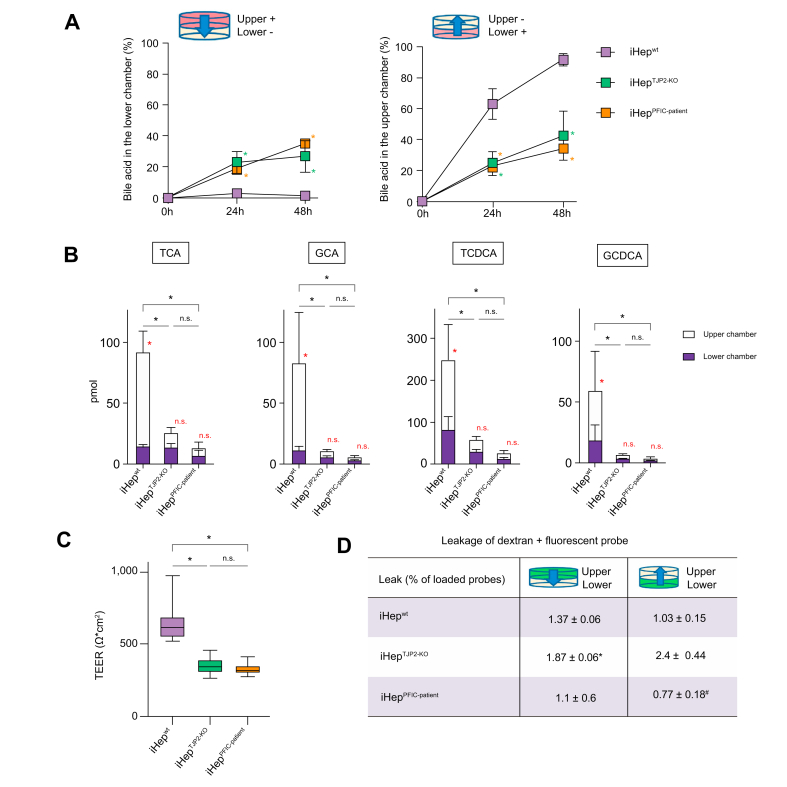
Impaired directional bile acid transport in TJP2-deficient iHep. (A) Bile acid (TCA) transport assay of iHep monolayer cultured in the Transwell system. The translocation of TCA from the upper (the apical domain) to the lower chamber (the basolateral domain) represents a bile acid leakage (the left panel), whereas the translocation of TCA from the lower to upper chamber represents an active bile acid transport (the right panel). The *y*-axis describes the percent TCA of the loaded TCA. iHep^wt^ showed minimal leakage of bile acid from the upper to lower chamber and active transport from the lower to upper chamber. However, iHep^TJP2-KO^ and iHep^PFIC-patient^ exhibited significantly more leakage and significantly reduced basolateral-to-apical transport. Mean ± SD, n = 3, where n corresponds to independent experiments, One-way ANOVA with a *post hoc* analysis comparing iHep^wt^ with iHep^TJP2-KO^, red ∗*p* <0.05, comparing iHep^wt^ with iHep^PFIC-patient^, blue ∗*p* <0.05. (B) Endogenous bile acid profiles were quantified by mass spectrometry. After exchanging culture medium, cells were incubated for 48 h, and the supernatants from the upper and lower chambers were collected separately. In iHep^wt^, all bile acid (TCA, GCA, TCDCA, and GCDCA) were excreted predominantly into the upper chamber, whereas in iHep^TJP2-KO^ and iHep^PFIC-patient^, the directional excretion was lost. iHep^TJP2-KO^ and iHep^PFIC-patient^ produced less amount of each bile acid. ∗*p* <0.05, mean ± SD, n = 3, where n corresponds to independent experiments. Red ∗ or ns: *t* test comparing the upper with lower chamber of each genotype. Black ∗ or n.s.: One-way ANOVA with *post hoc* analysis. (C) Two electrical probes are each placed in the upper and lower chambers to measure the TEER of the iHep^wt^, iHep^TJP2-KO^, and iHep^PFIC-patient^ cultured in the Transwell system. iHep^TJP2-KO^ and iHep^PFIC-patient^ exhibited significantly low TEER compared with iHep^wt^. Mean ± SD, n = 6, where n corresponds to independent experiments, One-way ANOVA with *post hoc* analysis, ∗*p* <0.05. (D) To evaluate paracellular leakage of monolayers in Transwell, cell-impermeable high-molecular-weight dextran conjugated with a fluorescent probe (Dex + AF647) was added to the upper or lower chamber. The leakage to the opposite chamber is measured after 24 h. iHep^TJP2-KO^ and iHep^PFIC-patient^ exhibited significant but negligible leakage compared with iHep^wt^. Mean ± SD, n = 3, where n corresponds to independent experiments, One-way ANOVA with *post hoc* analysis, ∗*p* <0.05 (iHep^wt^*vs*. iHep^TJP2-KO^), ^†^*p* <0.05 (iHep^wt^*vs*. iHep^PFIC-Patient^). GCA, glycocholate; GCDCA, glycochenodeoxycholate; iHep, iPSC-derived hepatocytes; iPSC, induced pluripotent stem cell; ns, nonsignificant; PFIC, progressive familial intrahepatic cholestasis; TCA, taurocholic acid; TCDCA, taurochenodeoxycholate; TEER, transepithelial electrical resistance; TJP2, tight junction protein 2.

The main functions of the tight junction at the bile canaliculi are to form a bile–blood barrier and to maintain bile acid transport in one direction (from sinusoid to canaliculus).^24^ It has been postulated that the bile–blood barrier in the liver of patients with TJP2 deficiency is compromised, thus leading to progressive cholestasis. To investigate the impacts of TJP2 deficiency in the bile–blood barrier and directional bile acid transport, we evaluated iHep on Transwell. The Transwell culture system is routinely used for quantification of the barrier function and directional transport between 2 biological domains.^25^ First, to quantify the bile–blood partition of hepatocytes, we loaded a bile acid, TCA, into the upper chamber and measured the TCA translocation into the lower chamber. In iHep^wt^, most of the loaded TCA was retained in the upper chamber, and a negligible percentage of TCA was detected in the lower chamber. In contrast, in iHep^TJP2-KO^ and iHep^PFIC-patient^, nearly 30% of the loaded TCA was detected in the lower chamber after 48 h, suggesting the loss of the bile canalicular function of bile acid segregation (Fig. 6A and Fig. S5A). To further test the directional bile acid transport from the basolateral domain to the apical domain, we loaded TCA into the lower chamber and measured it in the upper chamber. After 48 h, more than 90% of the loaded TCA were actively transported from the lower chamber to the upper chamber in iHep^wt^. In contrast, in iHep^TJP2-KO^ and iHep^PFIC-patient^, nearly 20% of the loaded TCA was detected in the upper chamber after 24 h and 40% after 48 h, suggesting the impaired of active directional bile acid transport in iHep^TJP2-KO^ and iHep^PFIC-patient^. The viability of iHep was comparable among conditions (Figs. S6A and S5B). To determine excreting direction of synthesised bile acid by iHep, we quantified the amount of endogenous bile acid in the culture medium of the upper *vs*. lower chamber using mass spectrometry. In iHep^wt^, all bile acid, TCA, glycocholate (GCA), taurochenodeoxycholate (TCDCA), and glycochenodeoxycholate (GCDCA), were excreted predominantly into the upper chamber, whereas in iHep^TJP2-KO^ and iHep^PFIC-patient^, the directional excretion was lost (Fig. 6B). iHep^TJP2-KO^ and iHep^PFIC-patient^ produced less amount of each bile acid. These results indicate that TJP2-deficient hepatocytes lose directional excretion of bile acid.

To delineate mechanisms of impaired bile segregation in TJP2-deficient hepatocytes, we quantified transepithelial electrical resistance (TEER). The TEER technique has been used routinely to assess the integrity of the tight junction between cells, where electrodes were placed in the upper and lower chambers to measure the electrical resistance (Fig. 6C and Fig. S5C). In our study, the TEER assay revealed that iHep^TJP2-KO^ and iHep^PFIC-patient^ had significantly lower resistance across the cellular monolayer compared with iHep^wt^, indicating the integrity and permeability of the monolayer have been compromised. To further test whether the integrity of the intercellular barrier is compromised by TJP2 deficiency, we incubated the upper and lower chambers with cell-impermeable fluorescence-conjugated dextrose polymers (Dex + AF647) and evaluated the paracellular leakage of the monolayer cultured between the chambers. iHep^TJP2-KO^ exhibited significantly more but negligible paracellular leakage from the upper to the lower chamber compared with iHep^wt^ but essentially comparable barrier function for larger molecules (Fig. 6D and Fig. S5D). The viability of iHep was comparable among conditions (Figs. S6B and S5E). Collectively, these results suggest TJP2 deficiency in iHep resulted in impaired directional bile acid transport coinciding with lower TEER, resembling the patient with cholestasis with *TJP2* mutation.

## Discussion

Modelling the bile canalicular function of human hepatocytes has been difficult because transporters on the canalicular membrane and the bile acid profile in human hepatocytes largely differ from other species. Despite the various previous attempts to recapitulate the cholestatic disease phenotype *in vitro*, it has been challenging to establish a reliable model to reflect the pathophysiology accurately. Previously, the genetic overexpression technique of intact or mutant transporters such as *ABCB4, ABCB11, ATP8b1,* in human embryonic kidney (HEK293T) cells, intestinal cancer cell lines (Caco-2 cells), or Madin–Darby canine kidney II (MDCKII) cells has been used to study progressive cholestasis with genetic mutations.[26], [27], [28], [29], [30], [31] However, such an approach has limitations in recapitulating the physiological transport machinery of human hepatocytes, making them less optimal for studies of liver pathophysiology and drug screening.^32^ Human iPSC technology provides an alternative path to build a novel model for cholestatic diseases.

Great efforts have been made in constructing ‘*in vitro* canaliculi’ in the field of drug-induced liver injury (DILI) for large-scale pharmacological testing of new therapeutic agents. Previous attempts include various sources of hepatocytes, such as the primary human hepatocytes, HepaRG cells, transdifferentiated hepatocytes, and mouse hepatocytes cultured in different systems, namely, hepatocytes in spheroids and in sandwich culture conditions, which give rise to polarised canaliculi-like structures.^33^^,^^34^ Recent advances in biomaterial allowed the canaliculi to develop between 2 adjacent mouse hepatocytes in the 3D microwell, and furthermore, in the presence of the specific extracellular matrix, even a single hepatocyte could establish a cellular polarity and canalicular structure.^35^ Based on the previous report, we established methods to culture iHep in the Matrigel sandwich and Transwell. When compared with a simple monolayer culture on plastic dishes, the Transwell culture system has the advantage of developing coherent hepatocyte polarity when the apical and basal membranes face each liquid phase. The Matrigel sandwich is feasible to scale up and has the potential for an imaging-based high-throughput drug screening assay. We have developed an image quantification system that enables us to screen the bile canalicular structure in the multiple-well plate. We have shown that our healthy iHep control demonstrates a similar ability in the transport of fluorescent tracer and bile canalicular structure to the primary hepatocyte control (Fig. S7). The 4 morphogenic parameters defined in our quantification assay accurately reflect the disruption of the bile canaliculi observed in the patients’ liver biopsy specimens. The Transwell system allows investigators to access both apical and basolateral domains easily and is a standard method to physiologically quantify barrier functions of a monolayer epithelium and biochemically detect target molecules. We used the Transwell culture system to quantify the barrier function of hepatocytes, aiming to address the function of the tight junction specifically. In our previous study, we reported that human iPSCs exhibited cellular polarity and directional bile acid transport when differentiated into hepatocyte-like cells on a permeable membrane of a 2-chamber system (Transwell).^13^^,^^15^ It is speculated that the microenvironment where cells interface with the liquid phase at both apical and basal domains promotes spontaneous polarisation of epithelialising cells. This limits scalability because Transwell requires fine manufacturing of the permeable membrane and culture well insets. To enable high-throughput drug screening assays on the Transwell culture system, an integration of microfluidics by microfabrication into the iPSC culture methods is critical.

By integrating a novel CRISPR technology, we were able to enhance the specificity of our method by generating isogenic pairs of iPSCs. We generated 2 patient-specific iPSC lines and 2 CRISPR-CAS9-edited iPSC lines with *TJP2* mutations. These engineered iPSC lines with deleterious mutations serve as complementary sets of TJP2-deficient cell lines by providing 3 pairs of isogenic iPSCs with controls. Having multiple isogenic iPSCs allows us to increase the reproducibility of our model system.

We report that 1 of our patients who carries a homozygous truncating mutation of p.R367X in *TJP2* gene showed a typical clinical presentation of PFIC. This is the first report of p.R367X mutation in *TJP2* gene for PFIC. The hepatocytes derived from this patient’s iPSCs exhibited comparable morphological and functional changes with the other hepatocytes with TJP2 deficiency (iHep^TJP2-KO^). This finding suggests that our system can recapitulate a biological consequence of newly found mutations and helps clinicians make a diagnosis of PFIC. An important next study is underway to test whether our system can recapitulate the biological effect of missense mutations in case the computational calculation is not able to predict the impact of genetic variants.

In our bile canalicular function assays, TJP2-deficient iHep exhibited abnormal direction of bile acid transport (from the canalicular domain to the sinusoidal domain), resembling hepatocellular cholestasis in patients with TJP2 deficiency. Reverse transport by deranged cellular polarity or paracellular leakage of bile acid can cause this abnormal bile acid transport. Morphological analysis of iHep with TJP2 deficiency demonstrated distorted cellular polarity and disruption of the apical membrane, which likely play an important role in the loss of directional transport of bile acid. TEER was low in TJP2-deficient hepatocytes, suggesting that impaired cell–cell tight junction also plays an important role in leakage of bile acid. A dextrose-conjugated fluorescent probe showed negligible leakage in TJP2-deficient hepatocytes, indicating that they maintain the barrier for larger molecules. This finding suggests that our Transwell system can model the defect in the function of the canalicular tight junction and revealed an important role of TJP2 in determining the cellular polarity of hepatocytes. This morphological feature of canalicular disruption was not observed in a liver specimen from a patient with BSEP deficiency, suggesting possible specific findings in TJP2-deficient liver (Fig. S8). However, the phenotype is likely overemphasised because of the *in vitro* environment of the Transwell system. The mechanical features of the membrane on Transwell, the monolayer culture conditions, and the lack of endothelial cells limit the ability of our system to recapitulate human livers with TJP2 deficiency. A multicellular coculture system with 3D biofabrication of the extracellular matrix will likely overcome the limitation.^36^

In the recent reports, Alb-Cre Tjp2-ko mice were born with healthy liver and exhibited hepatocyte injury only when fed with a high bile acid diet.^10^^,^^11^ In our study, TJP2 deficiency in human hepatocytes exhibits disruption of the canalicular membrane when differentiated into mature hepatocytes with bile acid synthesis, suggesting that bile acid plays a crucial role in TJP2 deficiency of human hepatocytes. Mouse hepatocytes synthesise more hydrophilic bile acid compared with human hepatocytes; therefore, the difference in the bile acid profile between a mouse and a human might cause the phenotypic difference in TJP2 deficiency in mice and humans.

In conclusion, we have shown that the human iPSC-derived hepatocyte model with TJP2 deficiency is capable of recapitulating the key functional links observed in patients, thus making it an attractive tool for drug screening and study for disease mechanisms.

## Financial support

AA was supported by 10.13039/100000002NIH grant P30 DK078392, Pilot & Feasibility Award (Sanger Sequencing, Research Pathology, Live Microscopy at the University of Cincinnati, Confocal Imaging, Transgenic Animal and Genome Editing, and Pluripotent Stem Cell Facility cores) of the Digestive Diseases Research Core Center in Cincinnati, the AASLD Foundation (Pinnacle Research Award), 10.13039/100011086North American Society of Gastroenterology Hepatology and Nutrition (NASPGHAN) Foundation (George Ferry Young Investigator Award), 10.13039/100010327Cincinnati Children’s Research Foundation (Procter Scholar Award), STR was supported by 10.13039/501100000265Medical Research Council (MRC) Clinician Scientist Fellowship Award (MR/L006537/1), UK. We thank PerkinElmer (UK) for supporting ZMT within the framework of the PE OneSource scheme and the Kings Medical Research Trust for supporting CZL with a studentship award.

## Authors’ contributions

Conceptualisation: AA, STR, RJT, HO, AM, OB, DD, YCH, CM, CZL, SSN. Investigation: AA, CZL, HO, SSN, XC, EK, KS, AF, YCH, CM, NLT, SJIB, AMS, LM, FX, MS, FS, OT, ZMT, HH. Writing—original draft: AA, CZL, SSN, HO. Writing—review and editing: AA, STR, RJT, CZL, SSN, JH, HH. Funding acquisition: AA, STR, RJT. Supervision: AA, STR.

## Data availability statement

The data that support the findings of this study are available from the corresponding author (A.A.), upon reasonable request.

## Conflicts of interest

STR discloses his shareholding and consultancy payments from DefiniGen Ltd. and Sana Bio. ZMT is employed by Perkin Elmer OneSource and seconded to the Stem Cell Hotel. The authors declare no conflicts of interest relevant to the study presented here.

Please refer to the accompanying ICMJE disclosure forms for further details.
