# Supplementary material for: Human iPSC-derived hepatocyte system models cholestasis with tight junction protein 2 deficiency
Source: JHEP Rep. 2022 Feb 1;4(4):100446. doi: 10.1016/j.jhepr.2022.100446 (PMC8904612; doi:10.1016/j.jhepr.2022.100446)
Supplement: Multimedia component 1 [file mmc1.pdf]

Human iPSC-derived hepatocyte system models cholestasis with tight junction protein 2 deficiency

Chao Zheng Li,Hiromi Ogawa, Soon Seng Ng, Xindi Chen, Eriko Kishimoto, Kokoro Sakabe, Hisamitsu Hayashi, Aiko Fukami, Yueh-Chiang Hu, Christopher N. Mayhew, Jennifer Hellmann, Alexander Miethke, Nahrin L Tasnova, Samuel J.I. Blackford, Zu Ming Tang, Adam M. Syanda, Liang Ma, Fang Xiao, Melissa Sambrotta, Filipa Soares, Oliver Baker, Davide Danovi, Richard J Thompson, S. Tamir Rashid, Akihiro Asai

Table of contents

Supplementary methods..2

Supplementary figures..4

Supplementary tables..12

Supplementary references..14

Supplementary methods

Generation of 2nd set of iPSC lines

We obtained skin fibroblasts from a patient (patient-2) who was diagnosed with TJP2 deficient PFIC by clinical genome sequencing, which revealed homozygous truncating mutations of p.Y261Sfs*50 (c.782delA) in exon 5 (Sup Fig S1A). We generated iPSCs from patient-2 ($iPSC^{PFIC\text{-}patient}$-2). Skin biopsy was taken, and the isolated research-grade fibroblasts were then reprogrammed using Epi5™ Episomal iPSC Reprogramming Kit at DefiniGen. The genotyping on generated iPSC showed preserved mutations. To mimic the truncating mutations of patient-2, we targeted the same exon 5 of *TJP2* to introduce a frameshift in iPSC lines derived from healthy donors, $iPSC^{wt}$-2 and 3 ($iPSC^{wt}$-2 was kindly provided by K. Eto and H. Nakauchi in Tokyo University and $iPSC^{wt}$-3 was provided by C&GT catapult UK). For isogenic exon 5 truncation of TJP2, after CRISPR editing and sub-cloning, we isolated an iPSC clone with homozygous truncating mutations, p.H162Mfs*145 (c.488delC) from $iPSC^{wt}$-2. As a result of the frameshift in exon 5, this $iPSC^{TJP2\text{-}KO}$-2 has premature stop codons at the same position 310 as $iPSC^{PFIC\text{-}patient}$-2 (Figure S1B). Another pair of isogenic iPSC was generated by introducing a large deletion into $iPSC^{wt}$-3 by using cas9 nuclease 3NLS and two different crRNA: TRACR duplexes (IDT) to cut >500 base pairs which include the exon 5 and a part of the upstream intron. The ribonucleoprotein complex was introduced through electroporation (NEPA21 125 V, 5ms, 4 mm cuvette) in a total volume of 100 µl buffer that included 1.0E6 cells, 1.6 µM RNP complex, and 0.8 µM cas9 nuclease. After electroporation, iPSC colonies were sorted onto 96 well plates for single-cell isolation by FACS. The survived colonies were expanded and evaluated for deletion by PCR-electrophoresis. The positive clones were then TOPO cloned by using the TOPO® TA Cloning® kit (Thermo Fisher) and, subsequently, confirmed by Sanger sequencing (Source BioScience plc). The PCR + TOPO cloning based screening identified a daughter iPSC clone with 691bp and 688 bp deletion ranging from the intron and the following exon 5 of each allele ($iPSC^{TJP2\text{-}KO}$-3), that induced alternative splicing and premature stop codon due to a frameshift, resulting in degraded mRNA of *TJP2* (Sup Fig S1C).

Immunostaining and imaging for iHep

Protocols for immunostaining in monolayer cells on the Transwell membrane were modified from previous reports[1]. In brief, cells were fixed with 4% paraformaldehyde at 4°C for 30 minutes, permeabilized with 0.5% Triton X100, and blocked with 5% donkey serum, then incubated with primary antibodies at 4°C overnight. The list of antibodies and dilution factors are described in Supplemental Table S1. For immunofluorescent histology, secondary antibodies with fluorescent probes were incubated at room temperature for 1 hour. The monolayer cells remained on the Transwell membrane throughout the process, and cover glasses were mounted onto the cell directly with mounting medium. Immunofluorescence microscopy imaging was performed using an Olympus microscope and DP71 camera (Olympus, Center Valley, PA) and Zeiss LSM710 confocal microscope (San Diego, CA). 3D image reconstruction of z-stack confocal images was generated using Imaris software (Bitplane, Concord, MA).

CDFDA functional assay in Matrigel sandwich

To evaluate the bile acid transport capacity and morphology of bile canaliculi in iHep, MRP2-specific substrate 5-(and-6)-carboxy-2′,7′-dichlorofluorescein (CDFDA) was supplemented into cells for 20 mins. Accumulation of fluorescent tracer (a metabolite of CDFDA) in bile canaliculi was then captured by CLS high content confocal imaging (Operetta CSL) and analyzed using ImageJ software. The images were first processed using global thresholding to filter out the noise and saturated signals. Next, size exclusion filtering was applied to remove imaging artifacts and to select biological relevant signals to determine the area, circularity, Feret's diameter, aspect ratio, roundness, and solidarity of the accumulated fluorescent tracer in bile canaliculi.

Bile acid transport assays

We seeded the dissociated cells onto the Transwell membrane after coating with Laminin. After confirming albumin secretion from iHep to the culture medium by ELISA, 50 μM of TCA (sodium taurocholic acid, Sigma) was added into the culture medium in the upper chamber for leakage assay. After 48 hours of incubation, the supernatant of the lower chambers was collected and stored until the analysis. The concentration of total BA in the culture supernatant was determined by the Diazyme TBA assay (Diazyme Laboratories) following the manufacturer's instructions. For the transport assay, TCA was added into the lower chamber and culture medium was collected from the upper chamber after 48 hours.

Trans-Epithelial Electrical Resistance (TEER)

Trans-epithelial electrical resistance was measured by EVOM2 (WPI) following the manufacturer's instructions. The culture media was replaced with buffer containing 118 mM NaCl, 23.8 mM NaHCO3, 4.83 mM KCl, 0.96 mM KH2PO4, 1.20 mM MgSO4, 12.5 mM HEPES, 5 mM glucose and 1.53 mM CaCl2 and incubated for 20 min before measurement.

Paracellular leakage assay

Alexa Fluoro 647 Dextrose 10,000 MW, AF647-10k (ThermoFisher), was used as a probe. The AF647-10k (25 μg/mL) was diluted in the culture medium in the upper or lower chamber. After 24 hours of incubation, the culture supernatant from the opposite chamber was collected, and the fluorescent intensity was measured by a plate reader. The concentration of the AF647-10k was determined by a calculation against the standard curve. The results were reported as a percentage of the loaded amount of AF647-10k in order to normalize the data for each well.

Cell viability assay

Cell viability was measured by the Cell-Titer-Glo kit (Promega). The viability was measured by ATP concentration of cell lysates. ATP concentration in $iHep^{wt}$ was used as a reference value.

Statistics

All in vitro experiments were performed at least in triplicate. Experimental values are expressed as mean ± SD, and statistical significance was determined by 2-tailed Student's t-test or by one-way ANOVA for comparison between 3 or more groups, followed by Bonferroni's multiple comparison post-hoc tests with a significance set at $p< 0.05$. Statistical analysis and graphic description were performed by GraphPad Prism (GraphPad Software).

human primary hepatocytes culture

Human primary hepatocytes in culture were purchased from GIBCO® Fresh Hepatocytes service (ThermoFisher, Waltham, MA) as described previously[2]. RNA was isolated from hepatocytes cultured on the plate for 24 hours.

Analysis of endogenous bile acid (TCA, TCDCA, GCA, GCDCA) concentrations by liquid chromatography-mass spectrometry (LC-MS)

After exchanging the culture medium, cells were incubated for 48 h. The supernatants from the upper and lower chambers were collected separately. Quantitative analysis of endogenous bile acid in the culture medium was carried out as described previously [2]. Briefly, mass quantification was performed by stable-isotope dilution LC-MS with electrospray ionization in single ion recording (SIR-MS) negative ion mode using a Waters TQ-XS triple quadruple mass spectrometer interfaced with Aquity UPLC system (Milford, MA). Quantification of each bile acid was achieved by interpolation of the area ratio of each bile acid to its corresponding stable-labeled analogue against a calibration curve of known concentrations of bile acid.

Supplementary figures

Supplemental Figure 1: 2nd set of iPSC lines

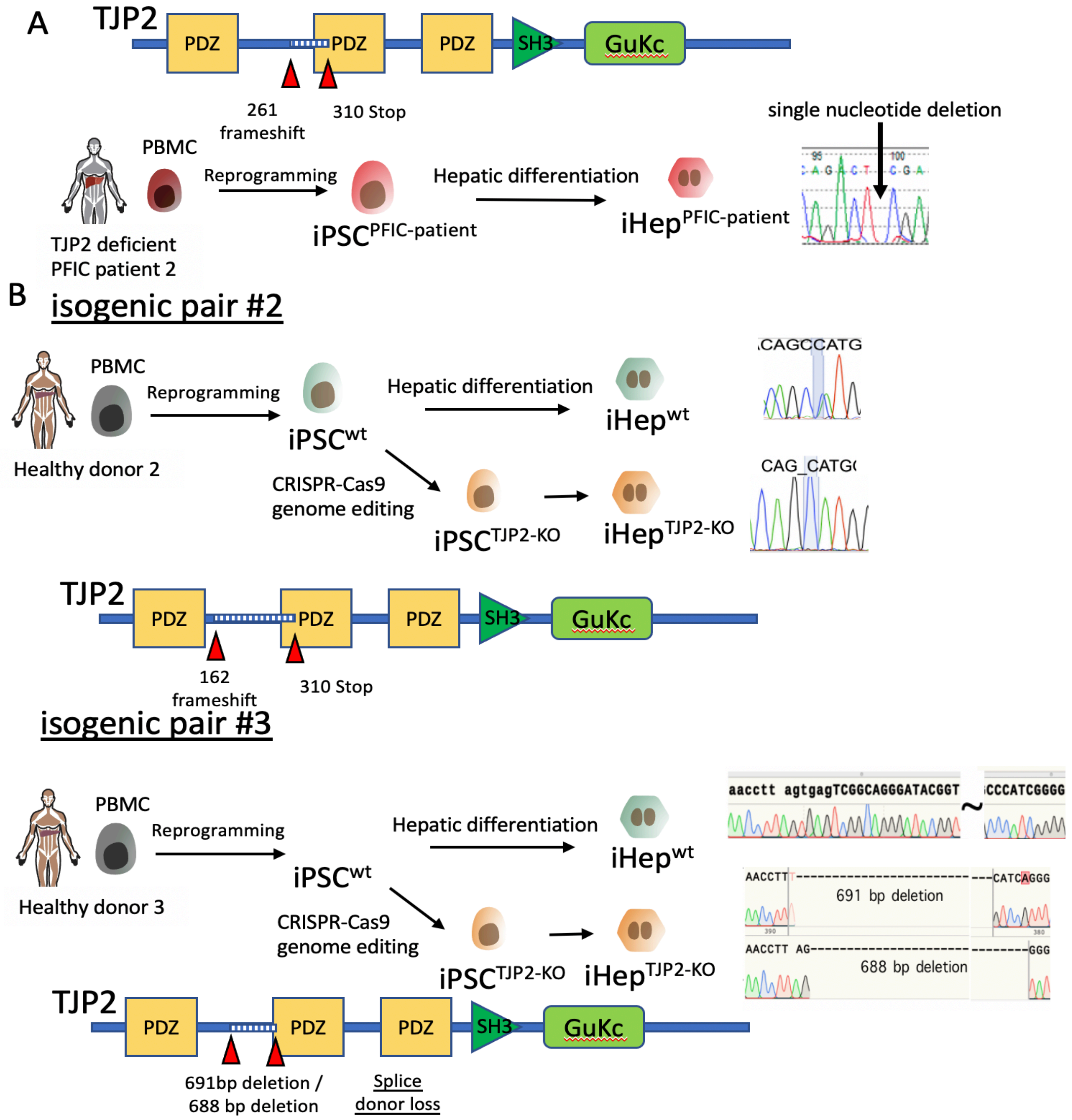

(A) Diagrams illustrating the location of TJP2 mutation (c.782delA) and frameshift and truncation of TJP2 (Stop at 310, exon 5). The dotted line indicates an altered amino acid sequence due to the frameshift. Sanger sequence confirmed the c.782delA in iHep differentiated from the iPSC derived from patient-2. (B) Schematic illustration of two sets of isogenic pairs of human iPSC lines. Two iPCS$^{TJP2\text{-}KO}$ lines were generated using the CRISPR-CAS9 genome-editing technology to introduce truncating mutations into *TJP2* gene at the same exon 5 in iPSC^{wt} lines derived from two healthy donors. The genotypes were confirmed by Sanger sequencing. Isogenic pair #2 was generated by introducing homozygous c.488delC, which results in a frameshift and protein truncation at 310 (c.488delC, p.H162M fs*148). Isogenic pair #3 was generated by introducing 691bp and 688 bp deletion into each allele of exon 5 (including intron between exon 5-6), which resulted in a loss of splice donor.

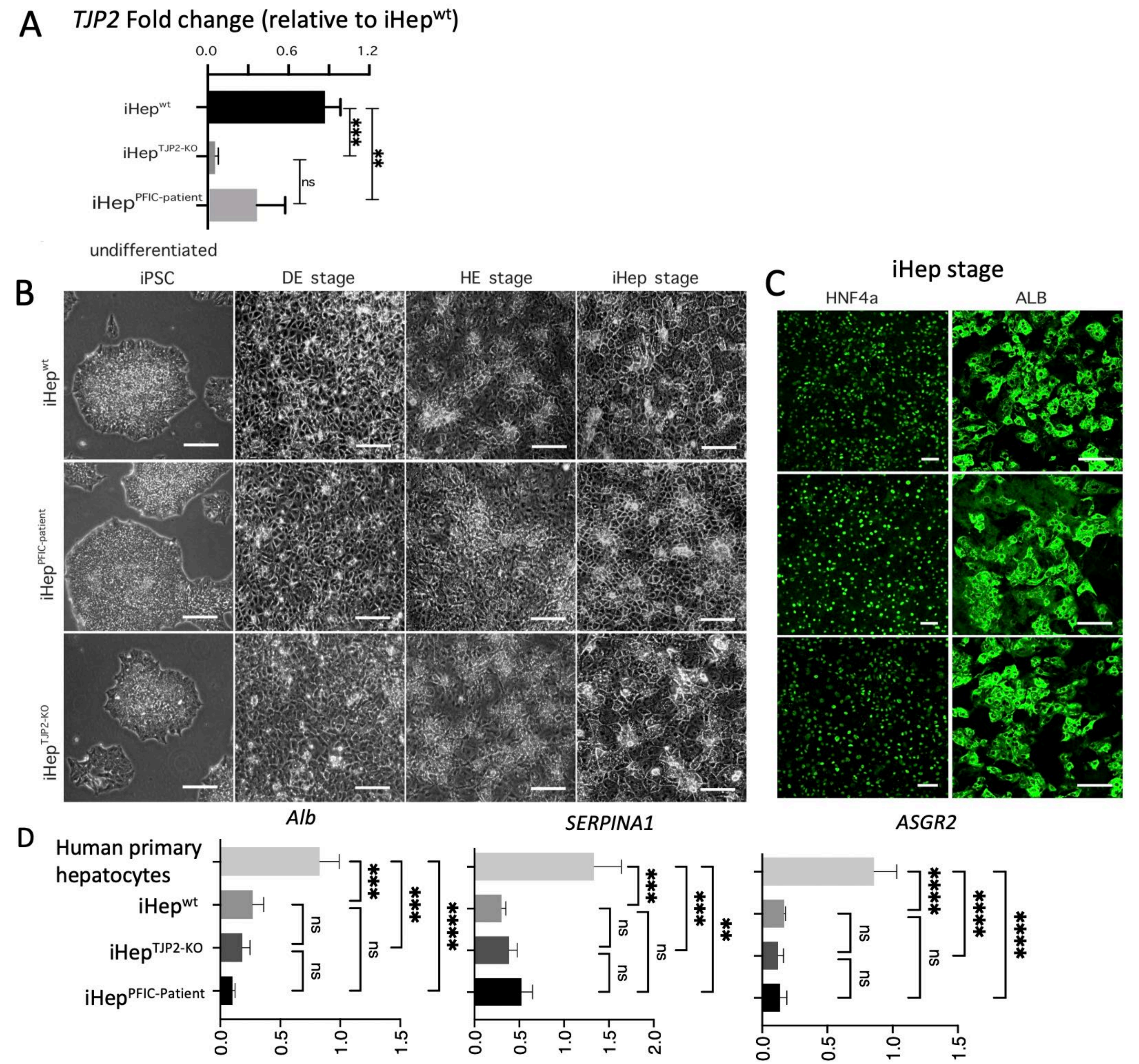

Fig. S2: Hepatic differentiation of the 2nd set of iPSC lines.

iPSC$^{TJP2\text{-}KO}$ and iPSC$^{PFIC\text{-}patient}$ (iHep$^{TJP2\text{-}KO}$ and iHep$^{PFIC\text{-}patient}$), compared to iHep^{wt}. Mean±SD, n=3, n corresponds to independent experiments, unpaired student t-test, **p<0.01, ***p<0.001, ns: nonsignificant. (B) Brightfield microscopy images reveal the morphological changes of each iPSC line at each stage of differentiation. Scale bar, 100µm. DE: definitive endoderm. HE: hepatic specification. (C) Immunofluorescence images detect the signature markers of hepatocytes (HNF4a: green, left, ALB: green, right), revealing a similar pattern of hepatic maturation in iHep^{wt}, iHep$^{TJP2\text{-}KO}$ and iHep$^{PFIC\text{-}patient}$. Scale 100µm (D) RT-qPCR gene expressions of hepatic genes (*ALB, SERPINA1, ASGR2*) are comparable among iHep^{wt}, iHep$^{TJP2\text{-}KO}$, and iHep$^{PFIC\text{-}patient}$. Relative gene expression was displayed as fold changes by -ΔΔCt method using 18S as a housekeeping gene and the expression level of human primary hepatocytes as a relative baseline. One-way ANOVA with post-hoc analysis, *p<0.05, **p<0.01, ***p<0.001, ns: not significant. Mean±SD, n=3, n corresponds to independent experiments.

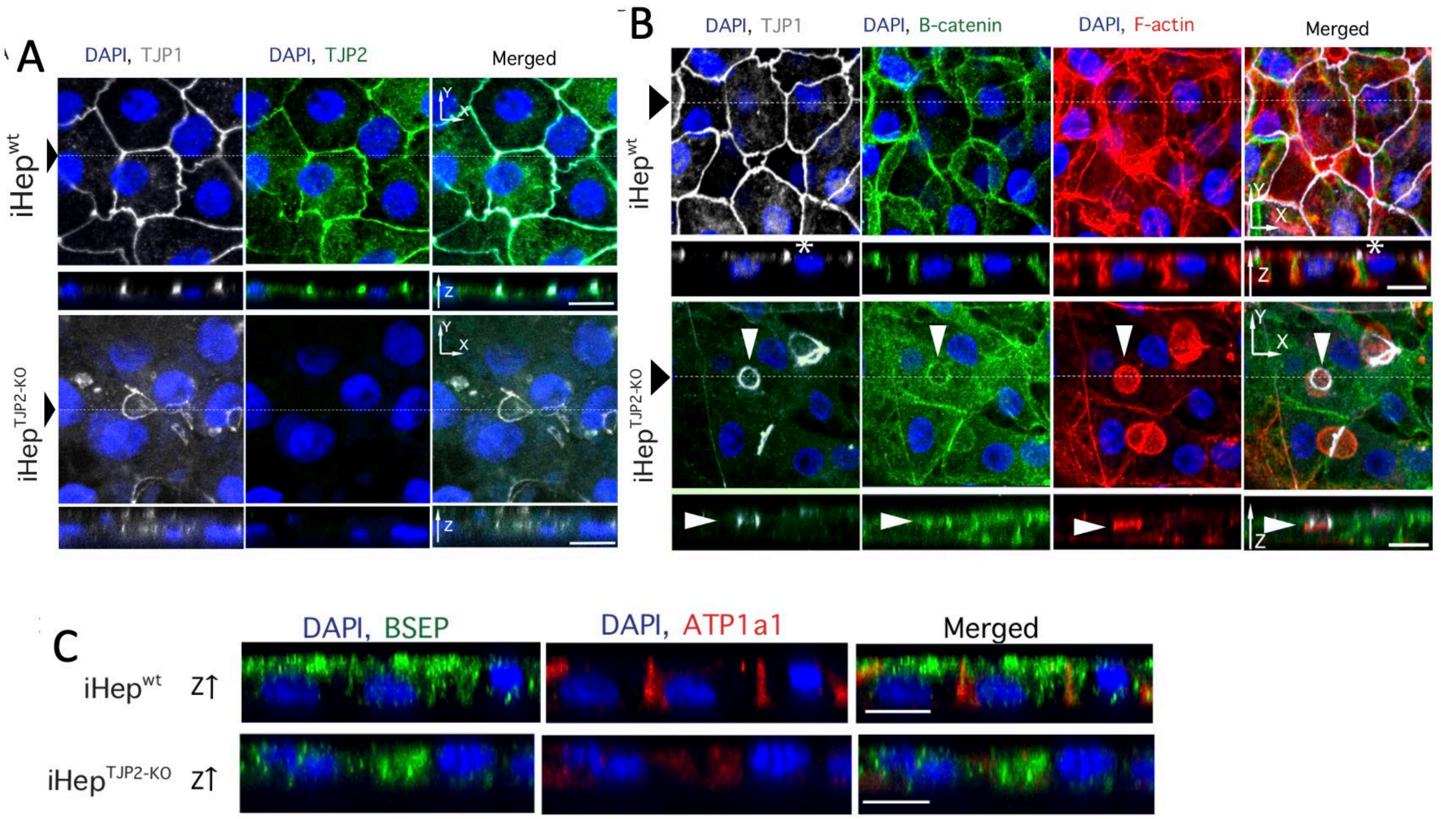

Fig. S3: Immunofluorescent staining of tight junction proteins, basolateral markers, and BSEP in isogenic pair #2. (A) Orthogonal view from different planes (x/y and x/z) of the confocal micrographs shows the expression of tight junction proteins, TJP1 (white) and TJP2(green), in iHep^{wt} and iHep$^{TJP2-KO}$ cultured in the Transwell system. In iHep^{wt}, TJP1 and TJP2 colocalize at the tight junction of adjacent cells. In iHep$^{TJP2-KO}$, TJP1 is detected in circular forms with random patterns, and TJP2 is not detected. The dotted white lines with black arrowheads indicate the position of Z-stacking. Scale bar, 10µm. (B) Orthogonal view from different planes (x/y and x/z) of the confocal micrographs show the TJP1 (white), beta-catenin (a basolateral membrane marker, green) and F-actin (red) of iHep^{wt} and iHep$^{TJP2-KO}$ cultured in the Transwell culture system. In iHep^{wt}, TJP1 is localized at the corner of cells at the junction of the apical and lateral membranes (white asterixis). F-actin is expressed on the apical and lateral membranes in iHep^{wt}. In iHep$^{TJP2-KO}$, the lateral localization of beta-catenin is disrupted. The circular pattern of TJP1 expression is overlapped with beta-catenin expression and partially with F-actin expression (white arrowheads). The F-actin surrounds spheroidal structures, and TJP1 demarcates them in ring patterns. The dotted white lines with black arrowheads indicate the position of Z-stacking. Scale bar, 10µm. (C) Reconstructed cross-section (x/z plane) of the confocal micrographs show the BSEP (an apical transporter in green) and ATP1a1 (a basolateral transporter in red) of iHep^{wt} and iHep$^{TJP2-KO}$ cultured in the Transwell culture system. BSEP is localized on the apical membrane, and ATP1a1 is localized on the lateral membrane in iHep^{wt}. The basal membrane is not visualized due to the optical interference of the plastic Transwell membrane. In iHep$^{TJP2-KO}$, BSEP and ATP1a1 localizations are disrupted. ATP1a1 expression is substantially reduced. Scale bar, 10µm.

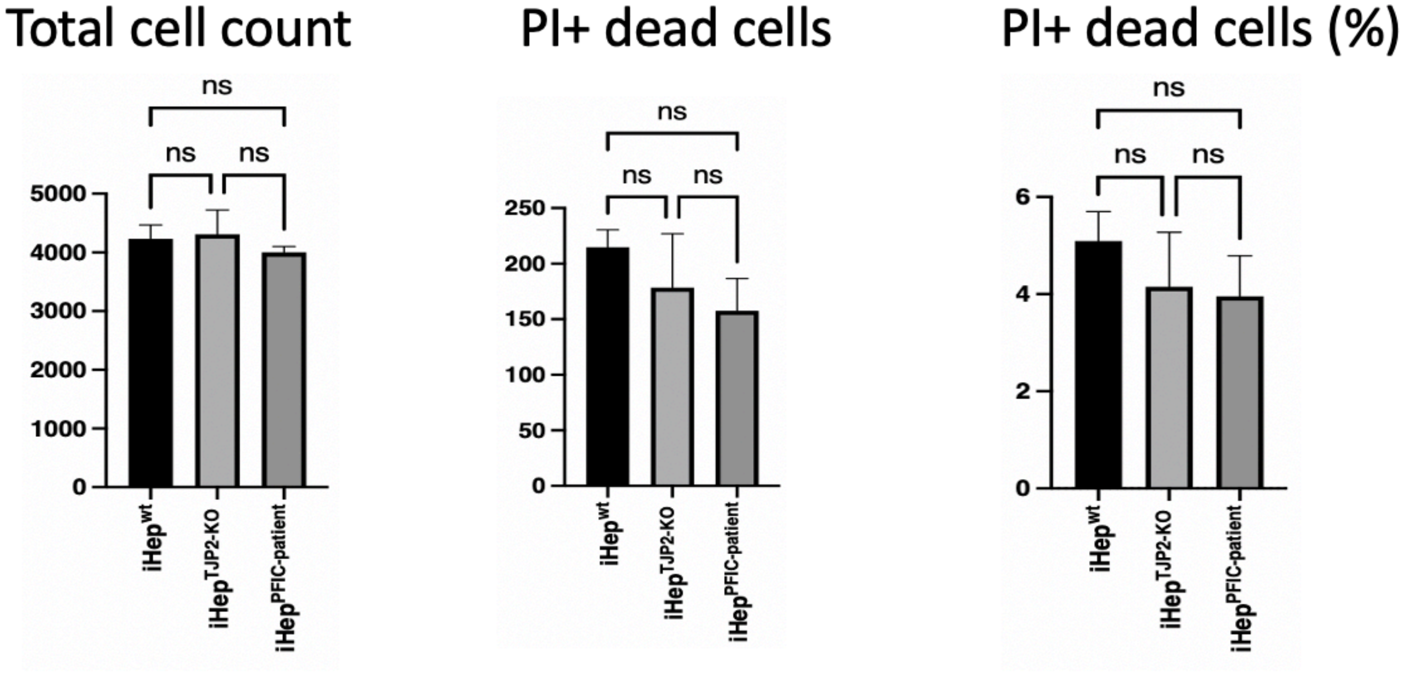

Fig. S4: viability of cells in the sandwich culture: The viability of cells in the sandwich culture was quantified by counting PI+ cells and normalized by the total cell counts in the image field. The percentage of dead cells was comparable in three cells lines. Mean±SD, n=3, n corresponds to independent experiments, One-way ANOVA with post-hoc analysis. ns: not significant.

A

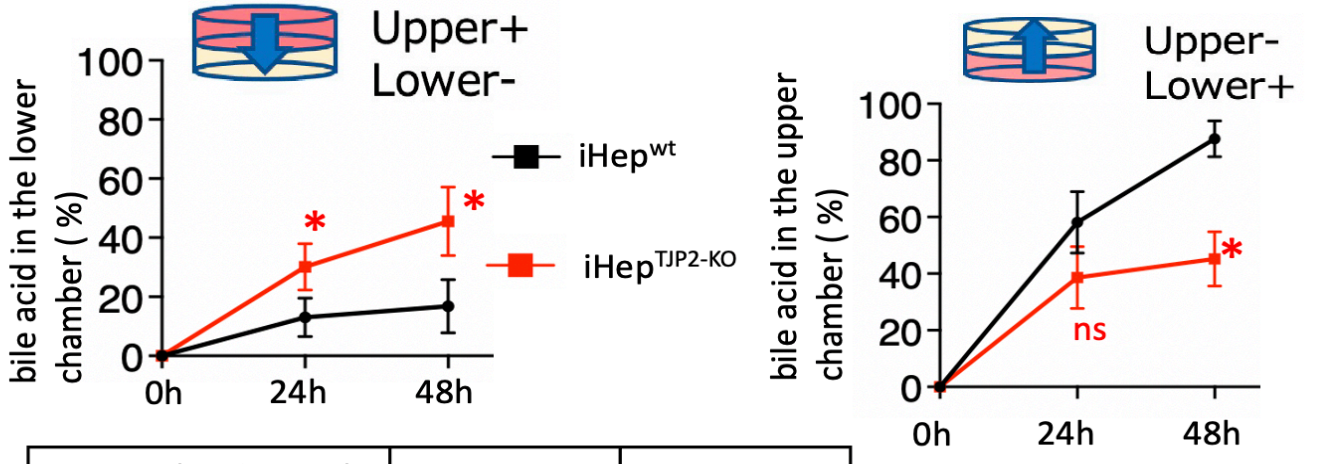

B

Viability (% of iHep^{wt})	Up to Low	Low to Up
iHep^{wt}	101.8 ± 4.26	99.8 ± 1.56
iHep$^{TJP2\text{-}KO}$	97.5 ± 5.36	103.6 ± 3.06

C

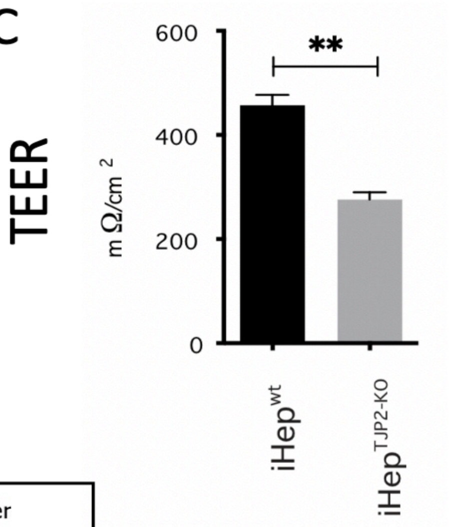

D Leakage of dextran + fluorescent probe

leak (% of loaded probes)	Upper Lower (↓)	Upper Lower (↑)
iHep^{wt}	0.96 ± 1.00	1.23 ± 0.54
iHep$^{TJP2\text{-}KO}$	0.58 ± 0.86	2.33 ± 1.44

E

Viability (% of iHep^{wt})	Upper Lower (↓)	Upper Lower (↑)
iHep^{wt}	104.3 ± 2.94	99.5 ± 3.39
iHep$^{TJP2\text{-}KO}$	99.33 ± 3.67	99.83. ± 3.25

Fig. S5: Barrier function for bile acid in isogenic pair #2: (A) The 2nd isogenic pair of iPSCs were used to reproduce the effect of TJP2 deficiency in bile acid (taurocholic acid: TCA) transport assay of iHep cultured in the Transwell system. The translocation of TCA from the upper (the apical domain) to the lower chamber (the basolateral domain) represents a bile acid leakage (the left panel), while the translocation of TCA from the lower to upper chamber represents an active bile acid transport (the right panel). The y-axis describes the percent TCA of the loaded TCA. iHep^{wt} showed a small leakage of bile acid from the upper to lower and active transport from the lower to upper. However, iHep$^{TJP2\text{-}KO}$ exhibited significantly more leakage and significantly reduced basolateral-to-apical transport. Mean±SD, n=3, n corresponds to independent experiments, t-test comparing iHep^{wt} vs. iHep$^{TJP2\text{-}KO}$, * $p<0.05$ (B) The cell viability after 48 hours of bile acid transport assays were comparable.

iHep^{wt} and iHep$^{TJP2\text{-}KO}$ cultured in the Transwell system. iHep$^{TJP2\text{-}KO}$ exhibited significantly low TEER compared to iHep^{wt}. Mean±SD, n=3, n corresponds to independent experiments, unpaired student t-test, ** $p<0.01$. (D) To evaluate paracellular leakage of monolayers in Transwell, cell-impermeable high-molecular-weight dextran conjugated with a fluorescent probe (Dex + AF647) was added to the upper or lower chambers, respectively. The leakage to the opposite chamber is measured after 24 hours. iHep$^{TJP2\text{-}KO}$ exhibited comparable leakage to iHep^{wt}. Mean±SD, n=3, n corresponds to independent experiments, unpaired t-test resulted in non-significance. (E) The cell viability after dextran leakage assays were comparable. Mean±SD, n=3, n corresponds to independent experiments, unpaired student t-test resulted in non-significance.

A

Viability (% of iHep^{wt})	Up to Low (not significant)	Low to Up (not significant)
iHep^{wt}	99.83 ± 2.79	100.15 ± 4.56
iHep$^{TJP2\text{-}KO}$	101.7 ± 8.24	96.89 ± 7.7
iHep$^{PFIC\text{-}patient}$	99.83± 2.31	106.83± 7.1

B

Viability (% of iHep^{wt})	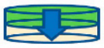 Upper Lower	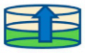 Upper Lower
iHep^{wt}	99.83 ± 3.66	101.7 ± 8.24
iHep$^{TJP2\text{-}KO}$	101.3 ± 6.89	98.33. ± 3.67
iHep$^{PFIC\text{-}patient}$	98.5 ± 5.96	97.5 ± 4.04

Fig. S6: (A) Cell viability was measured after 48-hour bile acid transport assays by the Cell-Titer-Glo kit (Promega). The viability was measured by ATP concentration of cell lysates. ATP concentration in iHep^{wt} was used as a reference value. Mean±SD, n=3, n corresponds to independent experiments, One-way ANOVA showed that values were not significantly different among the groups. (B) Cell viability of iHep after the experiments for the paracellular leak. Cell viability was measured by the Cell-Titer-Glo kit (Promega). The viability was measured by ATP concentration of cell lysates. ATP concentration in iHep^{wt} was used as a reference value. Mean±SD, n=3, n corresponds to independent experiments, One-way ANOVA showed that values were not significantly different among the groups.

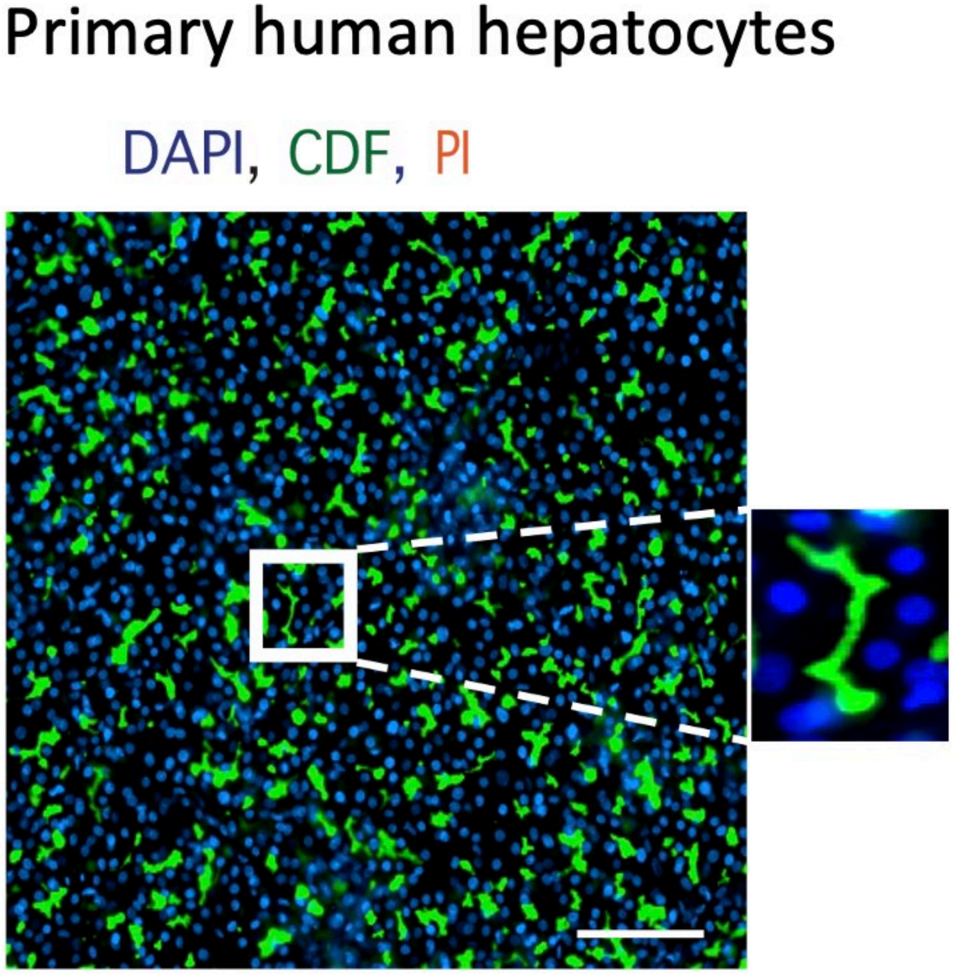

Representative confocal micrographs of human fetal liver cells show the transport and accumulation of a fluorescent tracer (CDF, a metabolite of CDFDA, green) representing the function and morphology of bile canaliculi of cells cultured in the Matrigel sandwich system. Dead cells are stained by propidium iodide (PI, red), and nuclei are stained by DAPI (blue). Fetal liver cells display chicken wire like the network of bile canaliculi by accumulating fluorescent tracer in canaliculi. Scale bar, 100µm

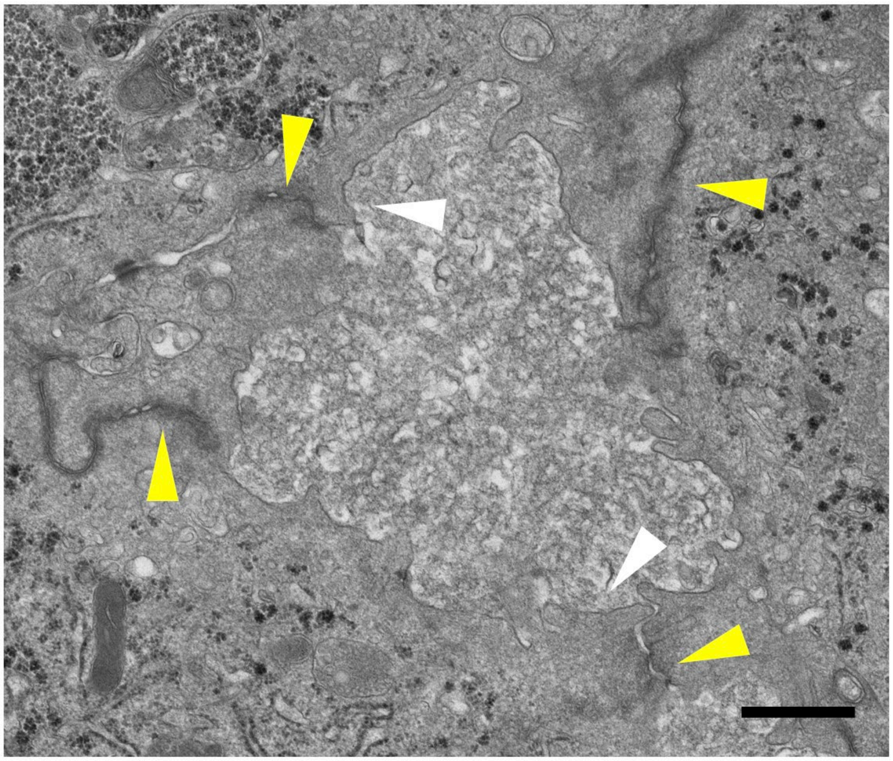

junction plaques (yellow arrowheads) are elongated. Apical disruption with inclusion bodies, seen in liver specimens with TJP2 deficiency, was not observed. Scale bars, 1µm.

Supplementary tables
Table S1

Secondary antibody list

	**Manufacturer**
Alexa Fluor 647 Goat anti-Rabbit	Thermo/Life Technologies
Alexa Fluor 488 Donkey anti-Rabbit	Jackson Immunoresearch
Alexa Fluor 488 Goat anti-Mouse	Jackson Immunoresearch
Alexa Fluor 647 Goat anti-Mouse	Thermo/Life Technologies
Alexa Fluor 488 Donkey anti-Goat	Abcam

Table S2

Primers for RT-qPCR list

**genes**	**priemrs 5' to 3'**
TJP2 EXON5f	TTGTGGTCAAGAGGCCCC
TJP2 EXON5r	GTTCAGCCGGCTCCTCTC
TJP2EXON4/7f	TCATTCGTTTGCAGTTCAGC
TJP2EXON4/7r	CTGGCTGTCTCTCAACACCA
ALBf	CGTCGAGATGCACACAAGA
ALBr	GATACTGAGCAAAGGCAATCAAC
ASGR2f	CGTGGGTGACAAGATCACAT
ASGR2r	GGGAAGTGCTTCAGATGGAA
SERPINA1f	CCGAAGAGGCCAAGAAACAG
SERPINA1r	GGTCTCTCCCATTTGCCTTT
CYP2E1f	GCTGTGGTGCATGAGATT
CYP2E1r	ACTACGACTGTGCCCTT

Table S3

The set code	iPSC ID for the study	TJP2 status	Original iPSC code	TJP2 genotype	TJP2 protein change	Age of donor (years)	Sex	Passage No.	Authentication test method
#1	iPSC$^{PFIC\text{-}patient}$	derived from patient #1 with TJP2 deficiency	N/A	Exon5<br>c.782delA	p.Y261S fs*50<br>Stop at 310	2	Female	25	Episomal clearance, Pluripotency marker by RT-qPCR, Hepatocyte differentiation
	iPSC^{wt}	Wild type	1383D6	WT	WT	36	male	40	Episomal clearance, Pluripotency marker by RT-qPCR, Hepatocyte differentiation
	iPSC$^{TJP2\text{-}KO}$	Isogenic iPSCs derived from control iPSC^{wt}	1383D6	Exon7<br>c.1099 C>T	p.R367X<br>Stop at 367	36	male	40	Episomal clearance, Pluripotency marker by RT-qPCR, Hepatocyte differentiation
#2	iPSC$^{PFIC\text{-}patient}$ -2	derived from patient #2 with TJP2 deficiency	N/A	Exon5<br>c.782delA	p.Y261S fs*50<br>Stop at 310	2	Female	35	Episomal clearance, Pluripotency marker by FACS and RT-qPCR, Hepatocyte differentiation
	iPSC^{wt}-2	Wild type	TkDA	WT	WT	unknown	Male	40	Episomal clearance, Pluripotency marker by RT-qPCR, Hepatocyte differentiation
	iPSC$^{TJP2\text{-}KO}$-2	Isogenic iPSCs derived from control iPSC^{wt}-2	TkDA	Exon5<br>c.488 delC	p.H162M fs145*<br>Stop at 310	unknown	Male	40	Episomal clearance, Pluripotency marker RT-qPCR, Hepatocyte differentiation
	iPSC^{wt}-3	Wild type	CGT-RCiB-10	WT	WT	55	female	32	Pluripotency marker by FACS and RT-qPCR, Hepatocyte differentiation..https://doi.org/10.1002/sctm.18-0084
	iPSC$^{TJP2\text{-}KO}$-3	Isogenic iPSCs derived from control iPSC^{wt}-3	CGT-RCiB-10	Exon5<br>691bp and 688bp deletion	Splice donor loss	55	Female	25	Episomal clearance, Pluripotency marker by FACS and RT-qPCR, Hepatocyte differentiation

Supplementary references

[1] Asai A, Aihara E, Watson C, Mourya R, Mizuochi T, Shivakumar P, et al. Paracrine signals regulate human liver organoid maturation from induced pluripotent stem cells. Development 2017. https://doi.org/10.1242/dev.142794.

[2] Hayashi H, Osaka S, Sakabe K, Fukami A, Kishimoto E, Aihara E, et al. Modeling Human Bile Acid Transport and Synthesis in Stem Cell-Derived Hepatocytes with a Patient-Specific Mutation. Stem Cell Rep 2021. https://doi.org/10.1016/j.stemcr.2020.12.008.
