# Supplementary material for: Human iPSC-derived hepatocyte system models cholestasis with tight junction protein 2 deficiency
Source: JHEP Rep. 2022 Feb 1;4(4):100446. doi: 10.1016/j.jhepr.2022.100446 (PMC8904612; doi:10.1016/j.jhepr.2022.100446)
Supplement: Multimedia component 2 [file mmc2.docx]

**Journal of Hepatology**

**CTAT methods**

Tables for a “Complete, Transparent, Accurate and Timely account” (CTAT) are now mandatory for all revised submissions. The aim is to enhance the reproducibility of methods.

- Only include the parts relevant to your study
- Refer to the CTAT in the main text as ‘Supplementary CTAT Table’
- Do not add subheadings
- Add as many rows as needed to include all information
- Only include one item per row

**If the CTAT form is not relevant to your study, please outline the reasons why:**

|  |
| --- |

- 1. **Antibodies**

| **Name** | **Citation** | **Supplier** | **Cat no.** | **Clone no.** |
| --- | --- | --- | --- | --- |
| HNF4a | Rabbit | Santa Cruz | sc8987 | N/A |
| Albumin | Goat | Bethyl | E80-129 | N/A |
| ATP1A1 | Mouse | Santa Cruz | 1/250 | C464.6 |
| ZO-1(TJP1) | Mouse | Life Technologies | 339100 |  |
| ZO-1(TJP1) | Rabbit | Thermo | 61-7300 |  |
| BSEP | Rabbit | Sigma | HPA019035 |  |
| Beta-catenin | Mouse | Santa Cruz | sc7963 | E-5 |
| TJP2 | Rabbit | Novusbio | NBP1-86850 |  |
| NTCP | Rabbit | Aviva | ARP42097 |  |
| Ecadherin | Goat | R&D systems | AF648 |  |
| Radixin (phosphor-ERM) | Rabbit | Cell Signaling Technology | 3141 |  |

- 1. **Cell lines (see Supplemental Table S3)**
  2. **Organisms**

| **Name** | **Citation** | **Supplier** | **Strain** | **Sex** | **Age** | **Overall n number** |
| --- | --- | --- | --- | --- | --- | --- |
| N/A | N/A | N/A | N/A | N/A | N/A | N/A |

- 1. **Sequence based reagents**

| **Name** | **Sequence** | **Supplier** |
| --- | --- | --- |
| Target Sanger sequencing | TJP2 gene | Source bioscience UK |

- 1. **Biological samples**

| **Description** | **Source** | **Identifier** |
| --- | --- | --- |
| Human fetal liver cells | Human fetal liver | Human Developmental Biology Resource of University College London |

- 1. **Deposited data**

| **Name of repository** | **Identifier** | **Link** |
| --- | --- | --- |
| **N/A** |  |  |

- 1. **Software**

| **Software name** | **Manufacturer** | **Version** |
| --- | --- | --- |
| Prism | GraphPad | 8 |
| Image J | FIJI | Java 1.8.0_172 |

- 1. **Other (e.g. drugs, proteins, vectors etc.)**

| 5(6)-Carboxy-2′,7′-dichlorofluorescein diacetate (CDFDA) | Merck | Cat: 21884-100MG | N/A |
| --- | --- | --- | --- |
| Propidium Iodide | ThermoFisher Scientific | Cat: P1304MP | N/A |

- 1. **Please provide the details of the corresponding methods author for the manuscript:**

| Akihiro Asai  3333 Burnet Ave. Cincinnati Ohio 45229 U.S.A.  +1 513-517-1013 (Tel)  +1 513-636-7805 (FAX)  Akihiro.asai@cchmc.org |
| --- |

**2.0 Please confirm for randomised controlled trials all versions of the clinical protocol are included in the submission. These will be published online as supplementary information.**

|  |
| --- |
